# Neurobehavioral, neuropathological and biochemical profiles in a novel mouse model of co-morbid post-traumatic stress disorder and mild traumatic brain injury

**DOI:** 10.3389/fnbeh.2014.00213

**Published:** 2014-06-23

**Authors:** Joseph O. Ojo, M. Banks Greenberg, Paige Leary, Benoit Mouzon, Corbin Bachmeier, Michael Mullan, David M. Diamond, Fiona Crawford

**Affiliations:** ^1^Roskamp InstituteSarasota, FL, USA; ^2^Research and Development Service, James A. Haley Veterans' HospitalTampa, FL, USA; ^3^Department of Life sciences, The Open UniversityMilton Keynes, UK; ^4^Department of Psychology, Department of Molecular Pharmacology and Physiology, Center for Preclinical and Clinical Research on PTSD, University of South FloridaTampa, FL, USA

**Keywords:** post-traumatic stress disorder, mild traumatic brain injury, mouse models, anxiety and social behavior, cognitive function, plasma and brain biomarkers

## Abstract

Co-morbid mild traumatic brain injury (mTBI) and post-traumatic stress disorder (PTSD) has become the signature disorder for returning combat veterans. The clinical heterogeneity and overlapping symptomatology of mTBI and PTSD underscore the need to develop a preclinical model that will enable the characterization of unique and overlapping features and allow discrimination between both disorders. This study details the development and implementation of a novel experimental paradigm for PTSD and combined PTSD-mTBI. The PTSD paradigm involved exposure to a danger-related predator odor under repeated restraint over a 21 day period and a physical trauma (inescapable footshock). We administered this paradigm alone, or in combination with a previously established mTBI model. We report outcomes of behavioral, pathological and biochemical profiles at an acute timepoint. PTSD animals demonstrated recall of traumatic memories, anxiety and an impaired social behavior. In both mTBI and combination groups there was a pattern of disinhibitory like behavior. mTBI abrogated both contextual fear and impairments in social behavior seen in PTSD animals. No major impairment in spatial memory was observed in any group. Examination of neuroendocrine and neuroimmune responses in plasma revealed a trend toward increase in corticosterone in PTSD and combination groups, and an apparent increase in Th1 and Th17 proinflammatory cytokine(s) in the PTSD only and mTBI only groups respectively. In the brain there were no gross neuropathological changes in any groups. We observed that mTBI on a background of repeated trauma exposure resulted in an augmentation of axonal injury and inflammatory markers, neurofilament L and ICAM-1 respectively. Our observations thus far suggest that this novel stress-trauma-related paradigm may be a useful model for investigating further the overlapping and distinct spatio-temporal and behavioral/biochemical relationship between mTBI and PTSD experienced by combat veterans.

## Introduction

There is a growing awareness of the consequences of mild traumatic brain injury (mTBI) and post-traumatic stress disorder (PTSD) in the veteran population of recent conflicts in Iraq and Afghanistan. mTBI is a complex clinicopathological entity, caused by external impact to the head or by a pressurized wave blast injury, resulting in a rapid rotational acceleration/deceleration of the brain in the closed skull of restrained occupants. Described as the major signature injury of these recent conflicts, approximately 18% of returning veterans have been diagnosed as having mTBI primarily due to exposures to combat related blast injuries from improvised explosive devices (Hoge et al., 2008).

Although the nature of a mTBI is such that there is a lack of overt brain damage such as hemorrhage or abnormalities visible from conventional computed tomography (CT) imaging scan, patients can eventually develop a complex clinical profile involving neurological symptoms such as: chronic headaches, dizziness, vertigo, memory-executive dysfunction, and loss of concentration (Bogdanova and Verfaellie, 2012). Neuropsychological symptoms can also arise due to the trauma surrounding the injury and involve insomnia, depression, irritability, impulsiveness, anxiety, apathy and aggression, resembling a cluster of PTSD like symptoms as clinically defined by the diagnostic and statistical manual of mental disorders (5th Edn.; DSM-5; American Psychiatric Association, 2013; see also Vanderploeg et al., 2012; Vasterling et al., 2012). Hoge et al. (2008) found that 44% of Iraq war returnees who experienced a loss of consciousness as a result of brain trauma also met the criteria for PTSD 3–4 months after deployment, compared to 27% of those reporting altered mental status, 16% with other injuries, and 9% with no injury. Combat related mTBI has also been demonstrated to approximately double the risk for PTSD (Schneiderman et al., 2008; Barnes et al., 2012; Bazarian et al., 2013). Over 300,000 service members (approximately one in five) to date are estimated to experience symptoms of PTSD. PTSD is triggered by an exposure to an intense trauma that threatens physical injury or death (with/without physical injury). Individuals respond to an acute traumatic experience or cues associated with the trauma with intense helplessness, fear or horror (DSM-5; American Psychiatric Association, 2013). The manifestation of this disorder is evident by the development and persistence (over many months) of symptoms such as anxiety, diminished extinction of conditioned fear, exaggerated startle response, and cognitive impairments (Milad et al., 2009; Norrholm et al., 2011). These re-experiencing and avoidance symptoms are further compounded by the addition of other co-morbidities, such as depression and substance abuse.

The clinical heterogeneity and large degree of overlap between mTBI and PTSD, including the diagnosis of both conditions either individually or co-morbidly, remains extremely challenging. Hitherto, most pre-clinical studies investigating response to psychological trauma and biomechanical head injury have remained largely separate focusing on both arms of the disorders in isolation, with only a few studies attempting to address the unique and overlapping features in a comorbid animal model (McAllister and Stein, 2010). This strongly underscores the need to develop a feasible and relevant translational pre-clinical model that extrapolates the uni- or multi-dimensional hallmarks of both disorders.

Of those that have exploited this vital relationship, intriguing results have been reported. Kwon et al. (2011) investigated the long-term consequences of unpredictable stress in the form of fox-urine exposure, loud noises and sudden cage movements, in combination with a blast injury induced by a compression-driven shock tube. The observations revealed pronounced and sustained impairment in memory function, neuronal and glial cell loss, inflammation and gliosis in blast injured mice exposed to stress. Similarly Elder et al. (2012) also examined rats with 3 repeated blast injuries, and reported long lasting (>40 days) PTSD-related behaviors, namely an impaired contextual fear conditioning, exaggerated acoustic startle response, and anxiety-like behaviors. Most recently, Klemenhagen et al. (2013) demonstrated a synergistic effect of repetitive concussive TBI and footshock stress on social and depressive-like behavior. These reported changes emphasize the need for mTBI and PTSD models, to recapitulate the sequence and hallmarks of the human condition. This field of preclinical research is in its infancy, and important elements in model design have yet to be fully explored. For example, the majority of studies have focused primarily on stress-trauma exposure in combination with mTBI(s) at separate timepoints. In this study we have tried to capitalize on combining a traumatic event with a mTBI, because it models a large population of PTSD patients (such as the combat veteran population) that experience an emotional trauma at about the same time that they also experience TBI (Barnes et al., 2012; Bazarian et al., 2013). In addition to this element, we have also tried to explore the effects of combining a mTBI on a background of continuous unpredictable repeated stress-trauma exposure as demonstrated by Kwon et al. (2011). Finally, although most studies have focused on rat models of PTSD (Zoladz et al., 2008, 2012), we have utilized a mouse model because of the potential future advantage of manipulating the genotype to explore factors that are implicated in the development and expression of PTSD.

In this study we have used our previously developed model of concussive injury, which has been extensively characterized from 24 h to 24 months post-injury (Mouzon et al., 2012, 2013; Ojo et al., 2013). These mice show evidence of memory dysfunction with repetitive mTBI, axonal injury, demyelination, white matter (corpus callosum) thinning, and glial activation. This concussive injury model was combined with a background of chronic PTSD paradigm involving unpredictable predator odor exposure (to trimethylthiazoline—TMT, a component of fox urine) under restraint, and a conditioned footshock stimuli. We report distinct and overlapping effects in neurobehavioral, neuropathological and biochemical profiles (in brain and plasma) in our newly developed model at an acute time-point (14 days post-exposure).

## Methods

### Animals: C57BL/6 mice

**Figure 1:**
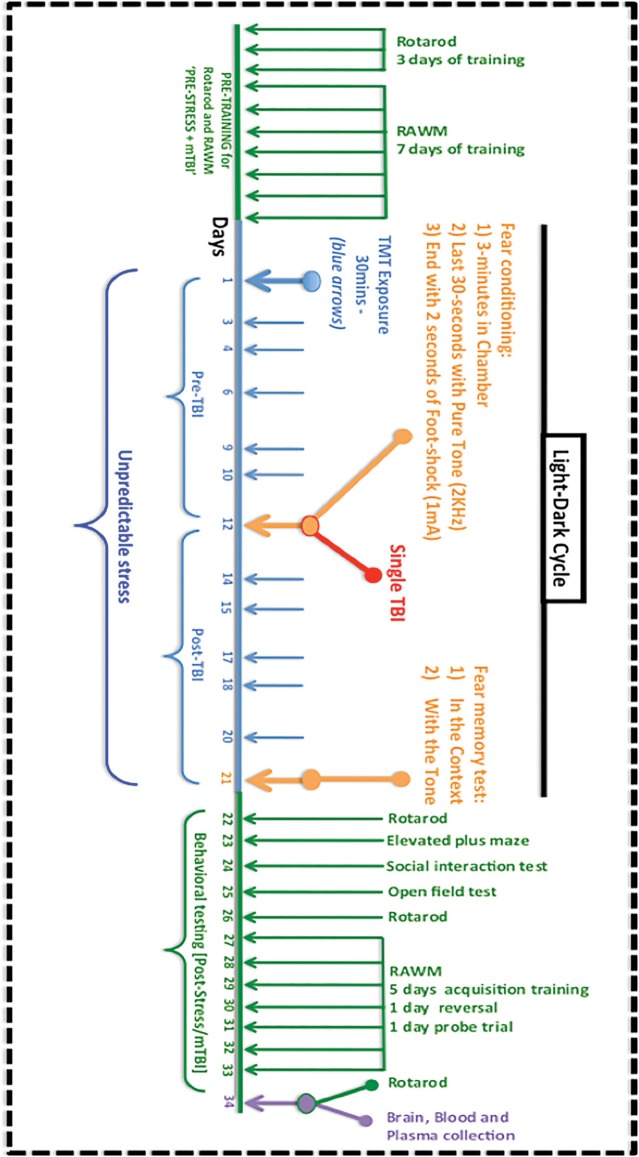
**Timeline and procedures for the experimental mTBI and PTSD paradigm**. Mice were pre-trained to obtain their baseline fall latency values on the rotarod test for motor co-ordination for 3 consecutive days, and this was followed by a 7-day acquisition training in the radial arm water maze (RAWM) test for spatial learning and memory. The 21 day experimental paradigm involved an exposure to TMT and restraint on 11 randomly assigned days (blue) at different times of the light-dark cycle. Around the midpoint, on day 12, mice underwent fear conditioning, which involved being placed in a conditioning chamber for 3 min, exposure to a 70 db auditory cue for the last 30 s, culminating with 2 s of a 1 mA foot-shock. Animals in the mTBI group were exposed to a single concussive head injury 1 h after the foot-shock, while under anesthesia. On day 21 animals were tested for their contextual and cued fear memory response. This was followed by a battery of behavioral tests for motor activity/coordination (rotarod), anxiety (elevated plus maze, open field test), social behavior and spatial learning and memory (RAWM). Brain tissue, plasma was collected for further studies.

Animals were purchased from Jackson Laboratories (Bar Harbor, Maine). All mice used in this study were male, aged 10–12 weeks (average 25–26 g) and housed in standard cages under a 12-h light/12-h dark schedule, at ambient temperature controlled between 22 and 23°C under specific pathogen free (SPF) conditions. Animals were given food and water *ad libitum* and maintained under veterinary supervision throughout the study. Mice were randomly assigned to each of the four experimental groups: mTBI alone, PTSD alone, mTBI+PTSD and control, with an *n* of 11–13 per group. Experiments were performed in accordance with OLAW and NIH guidelines under a protocol approved by the Roskamp Institute IACUC. For a timeline of all exposures and assessments see Figure 1.

### Traumatic stress exposure

Foot shock and unpredictable predator odor exposure(s) were chosen as the means of aversive stimuli (stressors) for inducing symptoms in accordance with each of the four clusters of the DSM-5 criteria involving: intrusion, avoidance, negative alterations in cognition and mood, and alterations in arousal and reactivity (American Psychiatric Association, 2013).

For the predator odor we used exposure to Trimethyliazoline (TMT) a component of fox feces/urine while animals were held immobile in a decapicone restrainer. This concept of immobilization was done in order to reproduce a mouse model analog to the persistent feeling of helplessness, numbness and loss of control, which are hallmark features of PTSD and critical in the expression of the stress response (DSM-5; American Psychiatric Association, 2013; Daskalakis et al., 2013). Within the 21-day stress exposure period, animals received TMT exposures at 11 random timepoints during the light-dark cycle, for a period of 30 min each session (see Figure 1). The element of sudden unpredictability/randomness of exposure was designed to disrupt any anticipation of the animal's perceptive instinct to the stressor. The long period of exposure (i.e., 30 min) was designed because human and animal studies suggest that prolonged exposure to trauma increases the likelihood of developing symptoms of PTSD (Zoladz et al., 2008; Zoladz and Diamond, 2013). The multiple exposures were designed to force the animals to re-experience the original stress response, therefore mimicking the repeated reliving of intrusive traumatic memories experienced by human patients (DSM-5; American Psychiatric Association, 2013; see also Zoladz et al., 2008). This is in agreement with combat soldiers who experience multiple traumatic experiences on the battlefield, and show increased likelihood of developing PTSD symptoms (see Vasterling et al., 2009; Daskalakis et al., 2013).

Fear conditioning was administered on day 12 at the mid-point of the 3-week stress paradigm. Briefly, this involved placing animals in a shuttlebox-chamber for 3 min, with a pure tone (70 db) introduced during the last 30 s. This tone intensity was chosen based on optimization from a previous experiment showing a minimal baseline (freezing) cued fear response to unstressed animals (Zoladz et al., 2008). At the end of the auditory cue, animals received a single 1 mA of uncontrollable/inescapable foot-shock for 2 s, and were allowed to recover for 1 min and then returned to their home cages. Animals in the mTBI only and PTSD-mTBI group received a single concussive brain injury 1 h after fear conditioning (see below for mTBI procedure).

For retention of contextual/cued fear memory animals were tested 9 days later. This involved exposing animals in the spatial context for 3 min and measuring the freezing response using a video tracking system (Ethovision; Noldus—Netherlands). A cued memory test was conducted 1 h after the contextual fear memory test, and involved placing animals in a novel environment for 3 min without the tone and a further 3 min with the tone. Freezing response was measured throughout the whole 6-min period. Post-behavioral battery tests were conducted on days 22–34 (see Figure 1).

### Experimental TBI procedure

The experimental TBI methods were performed as previously described (Mouzon et al., 2012). Briefly, mice were anesthetized with 1.5 L/min of oxygen and 3% isoflurane for 3 min. After shaving the injury site (around the anterior fontanelle), mice were transferred into a stereotaxic frame (Just For Mice—TM Stereotaxic, Stoelting, Wood Dale, IL) mounted with an electromagnetic controlled impact device (Impact-One TM Stereotaxic Motorized Impactor, Richmond, IL). The head/skull was positioned in the device, which prevented lateral movements as the impact was delivered. All mice were placed on a heating pad to maintain their body temperature at 37°C. A 5 mm blunt metal impactor tip attached to the electromagnetic motorized device was zeroed on the scalp and positioned above the sagittal suture midway before each impact using the NeuroLab controller. Upon satisfactory positioning, the tip was retracted and the depth adjusted to the desired level. The scalp was gently stretched by hand to restrict lateralization of the impact and to prevent the rod from delivering an inadequate trauma load at an irregular angle. Injury parameters were 5 m/s strike velocity, 1.0 mm strike depth, 200 ms dwell time and a force of 72 N″ (this paradigm was developed specifically as a mild injury with no skull fracture or subdural hemorrhage). Mice in the mTBI only and PTSD-mTBI group received one single concussive hit, while those assigned to the other groups received a single anesthesia of the same duration as their mTBI counterparts. After the impact was delivered, mice were then allowed to recover on a heating pad set at 37°C. Upon becoming ambulatory, mice were returned to their cages and carefully monitored for any abnormalities.

### Radial arm water maze (RAWM)

RAWM for spatial learning and memory was conducted as previously described by Park et al. (2008). This test was performed on two separate occasions pre and post-stress/injury. The experiment involved a radial arm water maze pool (120 cm diameter and 30–40 cm height), containing six swim paths, which extend from an open central arena, with an escape hidden platform beneath the end of one target goal arm. Briefly, animals were trained for 15 trials, which consisted of five blocks of 3 trials per day (lasting for a period of 5–7 days) to locate the hidden goal arm using visual cues located internally around the maze. Each trial lasted for 60 s. Those animals that entered into an incorrect arm were scored with an error (memory error score). The number of errors per block trial, across the entire length of the training session was recorded using a video-tracking system (Ethiovision, Noldus - Netherlands).

### Rotarod

Rotarod was conducted to evaluate sensorimotor coordination. First mice were tested pre-stress/injury for their baseline rotarod score i.e., ability to stay on a rotating rod for 3 × 5-min trials at accelerating speeds of 5–50 rpm (on 3 consecutive days). On days 2, 5, and 11 after the last TMT exposure, mice were tested again as previously (3 × 5 min trials) for their sensorimotor coordination (i.e., PTSD/mTBI related effects). The rotarod score, latency to fall (0–300 s) was calculated by averaging the mean of the three separate trials per day.

### Three chamber test for social interaction and novelty recognition test

The three chamber test quantifies the level of two social behaviors (i.e., social interaction and social memory or novelty recognition) between pairs of rodents. The test was conducted in a rectangular compartment that included a middle chamber with two doors leading to two separate (left and right) chambers, each containing a steel cage enclosure. After 5 min of habituation in the three chamber compartment, each mouse (experimental subject) was placed in the middle chamber and allowed to freely explore for 10 min, with the right chamber empty but an unfamiliar congener (Stranger I) held in the steel cage enclosure in the left chamber. Social interaction was determined by measuring the number of entries and length of time spent by the experimental subject exploring the chamber holding the unfamiliar congener vs. the empty chamber, and also the frequency and duration of intrinsic behaviors that compose social interaction such as, sniffing, allo-grooming, mounting. To measure social memory (or novelty recognition) a new novel stimulus mouse (stranger II) was placed in the enclosure in the previously empty right chamber and the previous unfamiliar mouse (stranger I) was retained in the same (left) chamber. The same parameters as above were measured to determine the preference of the experimental subject for stranger I and stranger II as an indication of social memory or novelty recognition.

### Elevated plus maze

The elevated plus maze is used to evaluate anxiety effects, based on a rodent's aversion of open spaces. The test consists of a plus shaped apparatus with two open and two enclosed arms, each with an open roof, elevated 50–70 cm from the floor. Each mouse was placed at the junction of the four arms of the maze, facing the open arm. The mouse was allowed to maneuver freely within the maze for 5 min; number of entries and duration in each arm (open/closed) were recorded with the aid of a video tracking system.

### Open field test

The open field test is also used to evaluate anxiety effects. This test was conducted in a large circular maze (120 cm diameter) setup in a brightly lit room. Animals were placed in the center of the maze and the number of entries/time spent in a predefined center zone and around the walls of the maze was recorded over a 15 min trial.

### ELISA

To obtain hematological blood specimens suitable for measurement of plasma glucocorticoid and cytokine(s) levels, animals were lightly anesthetized with isoflurane prior to euthanasia, and approximately 500 μl of blood was withdrawn into EDTA capillary tubes by cardiac puncture. Levels of glucocorticoids are very sensitive and can fluctuate based on the light and dark cycle of animals, environmental manipulations, and anticipation of food intake (McFall et al., 1992; Zoladz et al., 2012; Zoladz and Diamond, 2013). In our study, we obtained levels of corticosterone during the light cycle when levels are at their lowest and while animals were completely undisturbed. This timing was designed to avoid masking the potential rise in the circadian rhythm of corticosterone levels during the dark cycles with the true baseline levels of corticosterone. Samples were centrifuged at 5000 rcf for 3 min, and plasma samples (clear supernatant fraction) were flash frozen in liquid nitrogen and stored at −80°C. Plasma glucocorticoid levels were measured using an ELISA kit purchased from Life-sciences-Invitrogen, Garand Island, NY. Cytokine levels (IL-6, IL-1β, TNFα, IL-17A, IL-10, IFNγ) were determined using Bio-Plex Pro mouse Th17 panel ELISA kit (Biorad, Hercules, CA), as instructed by the manufacturers manual.

### Brain tissue preparation and western blotting

After the last post-behavior test on day 34, all animals were deeply anesthetized with isoflurane before being intracardially perfused by gravity drip with a heparinized phosphate buffered solution (PBS) pH-7.4 for 3 min. One hemisphere was collected and flash frozen in liquid nitrogen and kept at −80°C for antibody based/biochemical analyses and the other hemisphere was post-fixed in 4% paraformaldehyde (PFA) for histological/immunohistochemical analyses.

For western blotting analyses, each half brain was homogenized in 750 ul PBS (pH 7.4) containing proteinase inhibitor cocktail using a dounce homogenizer. Homogenized samples were spun in a centrifuge at 15,000 rpm for 3 min. Tissue supernatants were collected and denatured by boiling in Laemmli buffer (Bio-Rad, CA, USA) and resolved onto 4–20% gradient polyacrylamide gels (BioRad, CA, USA). After electrotransferring onto polyvinylidene difluoride membranes, western-blots were immunoprobed for different brain specific markers of neurodegeneration: neurofilament L (NFL), phospho-tau serine-202 (CP13), glial fibrillary acidic protein (GFAP), and intracellular cell adhesion molecule -1 (ICAM-1). NFL (rabbit polyclonal anti-NFL, Millipore, Billerica, MA), CP13 (Peter Davies), ICAM-1 (mouse anti-ICAM-1, Abcam, Cambridge, MA), and GFAP (rabbit anti-GFAP, Dako, Carpentaria, CA). An anti-actin mouse monoclonal antibody (Chemicon, CA, USA) was used as a reference antibody to quantify the amount of proteins electrotransferred, and NFL/actin, CP13/actin, ICAM-1/actin, and GFAP/actin signal intensity ratios were quantified by chemiluminescence imaging with the ChemiDocTM XRS (Bio-Rad, CA, USA).

### Immunohistochemistry

Hemispheres obtained from each mouse after transcardial perfusion with PBS were post-fixed for >5 days in 4% paraformaldehyde, dehydrated in sucrose and weighed. 50 um thick coronal sections were cut using a cryostat set at −17°C (Leica, CM300, Buffalo Grove, IL) throughout the whole rostro-caudal extent of the cortex, hippocampus, and amygdala and stored in a 48 well-plate containing cryoprotectant—30% sucrose and 30% ethylene glycol. A 1 in 10 series of 5 separate sections through the regions of interest guided by known bregma co-ordinates, was collected for immunohistochemical (IHC) assessment of different markers. Briefly, standard IHC involved rinsing free-floating sections in deionized water and subsequently incubating sections at room temperature in a solution of endogenous peroxidase blocking solution, containing 3% hydrogen peroxide and 10% methanol diluted in deionized water for 30 min. After rinsing, sections were treated with target retrieval citrate buffer solution (pH 6) for 30 min at 90°C (if recommended) to induce heat mediated antigen retrieval. Further incubation with either protein block serum free solution (Dako Carpinteria, CA) or 0.01% Tween 20 in PBS containing 10% normal serum solution to which the secondary antibody was raised (Vectors lab, Burlingame, CA), was carried out for a period of 1 h at room temperature depending on the antibody used.

Sections were stained in entire batches with primary antibodies made up in either antibody diluent background reducing agent from DAKO or 1% normal serum. Primary antibodies used were raised against:

- GFAP (rabbit anti-GFAP, 1:25,000, Dako, Carpinteria, CA) for astrocytosis.
- CD45 (rat anti-mouse CD45, 1:1000, Serotec, Raleigh, NC) for microgliosis.
- Doublecortin, DCX (rabbit anti-DCX, 1:1000, Abcam, Cambridge, MA) for immature neurons.
- c-Fos (rabbit anti-cFos, 1:1000, Millipore, Billerica, MA) immediate early gene cFos.
- Laminin (rabbit anti-Laminin, 1:25, Sigma, St Louis, MO) for blood vessels.
- Synaptophysin (rabbit anti-SYN, 1:50, Dako, Carpinteria, CA) for synapses.
- ICAM-1 (rabbit anti-ICAM-1, 1:800, Santa Cruz, Dallas, TX) for inflammation.
- Iba1 (goat polyclonal anti-Iba1, 1:2500, Abcam, Cambridge, MA) for microglia.

After overnight incubation with the relevant primary antibodies, sections were rinsed with PBS, transferred to a solution containing the appropriate secondary antibody (from the Vectastain Elite ABC Kit, Vector Lab, Burlingame, CA) diluted in 0.01% Tween 20 containing 1% normal serum for 1 h, and further incubated with avidin- biotin-horseradish peroxidase solution (Vectastain Elite ABC kit; Vector Lab, Burlingame, CA) for a further hour. Immunoreactivity was visualized with 3,3″-diaminobenzidine (DAB) chromogen. Development with the chromogen was timed and applied as a constant across batches to limit technical variability (in immunodetection) before progressing to quantitative image analysis. The reaction was terminated by rinsing sections in distilled water. Finally, mounted sections counterstained in hematoxylin, were progressed through a graded series of alcohols (dehydrated), cleared in xylene and coverslipped with permanent mounting medium. Negative control sections were included whereby the primary antibody was omitted and replaced either with blocking agent or biotinylated secondary antibodies alone. Immunoreacted sections were viewed using an Olympus BX63 light microscope with a high resolution DP72 camera (Olympus, Center Valley, PA).

### Image analysis

Immunoreactivity for (GFAP, IBA1) cell markers was measured by quantitative image analysis (optical segmentation). Rigorous staining protocols were applied to ensure consistency of immunostaining and accuracy of image analysis. This procedure was performed by blinded assessment (with each slide analyzed blind with respect to marker or animal group). Firstly, a survey of immunoreacted tissue sections was performed independently to verify specific immunoreactivity in each series (~1 in 10) of sections that was subsequently progressed to quantitative image analysis. Briefly, non-overlapping RGB (red, green, blue) images were digitally captured randomly within the defined areas from each section (comprising an average of 5 sections per animal for each region and marker), providing a randomized-systematic survey of each region throughout the area of interest for each animal. A total area of 0.65 mm^2^ microscopic field (captured at ×60) was analyzed for each region per animal. For both cellular markers we analyzed the corpus callosum (CC), parietal cortex region beneath the injury site, hippocampus (CA1–CA3), the basolateral amygdala, and the medial and periventricular hypothalamus (in regions bound by the bregma co-ordinates −0.94 to −2.75). Due to the limited number of serial sections we were unable to analyze the distinct subnuclei within the hypothalamus separately, focusing primarily on nuclear regions primarily involved in controlling aspects of stress-related endocrine functions. We omitted the lateral hypothalamus because it has a lesser role in stress-related endocrine functions.

Immunoreacted profiles that were optically segmented were analyzed using FIJI-Image J morphometric image analysis software. A semi-automated RGB color threshold function was employed to determine the optimal segmentation (threshold setting) of immunoreactivity for each antibody. Deconvolution was performed on counterstained sections to subtract the background counterstain. Immunoreactive profiles discriminated in this manner were used to determine the specific mean immunoreactive area of staining. Data were separately plotted as the mean percentage area of immunoreactivity per field (denoted “% Area”) ± SEM for each region and grouping.

To determine changes in doublecortin immunoreactive (ir) cell counts in the DG, a 1 in 10 series averaging >5 sections was chosen in a random systematic manner through the region of interest. Doublecortin-ir cell somata were easily discriminated. An average of the total number of DCX+ cells in the dentate gyrus (DG) of the hippocampus of each of the five separate sections containing the hippocampal portion was determined.

### Volumetric estimates

The volume of the right hippocampus as guided by bregma co-ordinates (−0.94 to −3.28 mm) and the corpus callosum (bound by bregma co-ordinates −0.94 to −2.30 mm) was determined by quantitative light microscopy using the Cavalieri method as described elsewhere (Ojo et al., 2011). In brief, rostrocaudal sections from the extent of the right hippocampus of each animal (taking every sixth serial section) were mounted onto superfrost plus slides, after washing with 0.1 M PB at pH 7.4 to remove the cryoprotectant storage solution. An average of 12–15 sections were collected per animal. Mounted sections were air-dried and stained with a solution of 0.1% cresyl fast violet (Tedpella, Reading, CA) and were viewed at low magnification using a DP72 digital camera attached to a motorized Olympus BX63 digital photomicroscope (Olympus, Center Valley, PA). Analyses were carried out blind and were made via the Cell SENS Olympus software package. Digital images were captured electronically and the boundaries of the total hippocampal compartment and corpus callosum in the region of the injury site (bound by bregma co-ordinates −0.94 to −2.30 mm) were digitally outlined on each section from the series of rostro-caudal sections of the right hemisphere. For each animal, the total volume of the right hippocampus and corpus callosum (bound by bregma co-ordinates −0.94 to −2.30 mm) was subsequently derived by multiplying the calculated total surface area by the mean distance between the series of sections. Data are presented as mean volume (in mm^3^) ± SEM.

### Statistical analysis

Data were tested for normal (or Gaussian) distribution using the D'Agostino and Pearson omnibus K2 normality test for skewness and kurtosis. Homogeneity of variance was tested using the Browns-Forsythe and Bartlett's test. An alpha criterion level of *P* < 0.05 was set for all statistical significance. Analyses were performed using the Graphpad prism software (LaJolla, CA).

#### Neurobehavior

Overall mean percent freezing values during the contextual fear memory test, mean errors on the final day of RAWM training and mean time in center zone during open field test were analyzed using One-Way analysis of variance (ANOVA). A regular Two-Way ANOVA was used to analyze the overall mean percent freezing values during the cued fear memory test with the two subject factors of cued-tone and exposure groups/paradigms. Repeated measures-Two Way ANOVA was used to analyze contextual fear memory, cued fear memory, RAWM memory errors, rotarod, open field center zone entries and motor activity data, with the two subject factors of “group exposure” and a repeated within subject factor of “time blocks” across the trial or training session of each test. Tukey's *post-hoc* test was performed when appropriate. Elevated plus maze data did not fit the normal (or Gaussian) distribution and were analyzed with the non-parametric Kruskal Wallis One-Way ANOVA test followed by Dunn's correction for multiple testing. Social interaction and social novelty recognition data for each group exposure were analyzed using the Mann Whitney *U*-test for the dependent measures of time spent between stranger I and stranger II chambers.

#### Biochemistry and histopathology

Following normality testing, biochemistry data (corticosterone, cytokine profiles, brain injury biomarkers) and histopathological data (brain weight, hippocampal and corpus callosum volumes and doublecortin cell counts) were analyzed using the non-parametric Kruskal Wallis One-Way ANOVA test followed by Dunn's correction for multiple testing where appropriate. GFAP and IBA1 immunoreactivity data (per brain region) were analyzed using One-Way ANOVA.

## Results

### Contextual and cued fear memory response and spatial learning and memory performance in a mouse model of mTBI-PTSD

**Figure 2:**
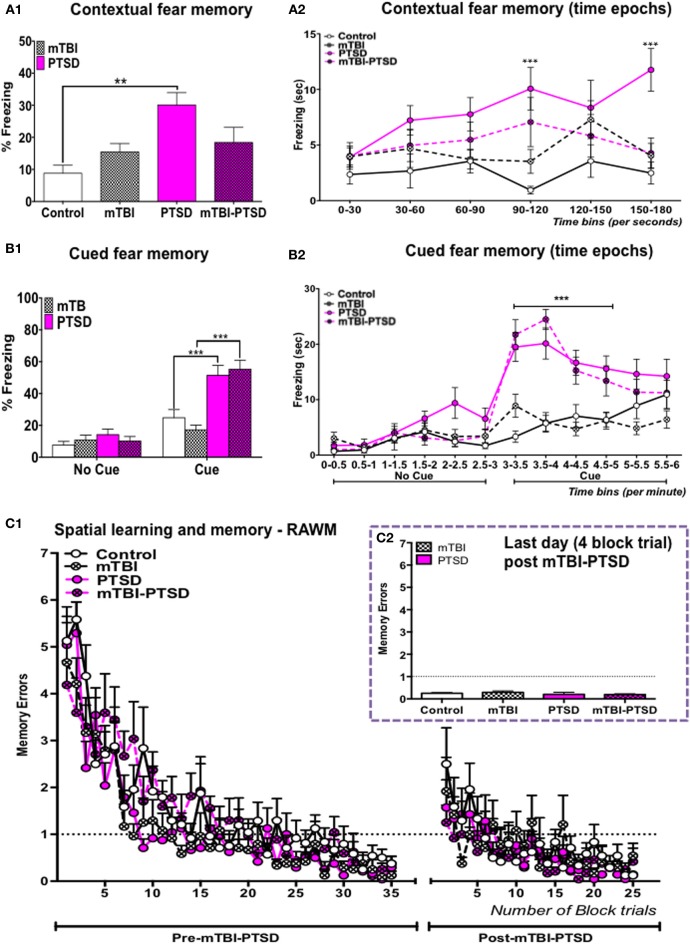
**Contextual, cued fear, and spatial memory**. PTSD animal group showed a significant increase in their (%) freezing responses to both the context **(A1,A2)** and the auditory cue **(B1,B2)**, compared to control animals. mTBI inhibited the retrieval of the contextual fear memory response in the mTBI-PTSD group **(A1,A2)**, there was no effect of mTBI on the cued fear response in the mTBI-PTSD group **(B1,B2)**. No significant effect was seen in the fear memory response from the mTBI only group compared to the control group in both the context and with the auditory cue (*P* > 0.05; *n* = 8–13). All animals performed equally in the pre-training session in the RAWM test, reaching the training criteria of one-memory errors by the end of the 7-day acquisition training session **(C1)**. Two-way analysis with repeated measures showed no main effect of exposure (and their interaction with time; *P* > 0.05) with either PTSD, mTBI or their combinations on spatial learning and memory of a pre-learned task **(C1)** (*n* = 8–9). No effect was seen on mean memory error during the last day of the trial **(C2)**. Data are presented as mean ± SEM. X-axis in **(A2,B2)** represent 30 s and 0.5 min (time) epochs over the entire length of the trial. Data in **(A1,C2)** were analyzed using One-Way ANOVA. Data in **(B1)** were analyzed using a regular Two-Way ANOVA. Data in **(A2,B2,C1)** were analyzed using a repeated measures-Two Way ANOVA. Tukey's multiple comparisons *post-hoc* test was performed in all cases. Asterisks denote statistical significance as follows: ^**^P < 0.01; ^***^P < 0.001.

Interactions between mTBI and PTSD were examined on contextual and cued fear memory response 9 days after fear conditioning induced by a 1 mA footshock and 70 db auditory cue. One-Way ANOVA showed a significant main effect across the study groups in the context over the 3 min trial session [*F*_(3,30)_ = 5.875; *P* = 0.003; Figure 2A1). This effect was also observed when the data for each trial session were broken into 30-s blocks. A repeated Two Way ANOVA showed a significant main effect across the study groups [*F*_(3, 30)_ = 5.432; *P* = 0.004], no significant main effect was observed on the variance over time blocks [*F*_(5, 150)_ = 2.207; *P* = 0.057], and a significant interaction between the study groups and variance over time blocks was observed [*F*_(15, 150)_ = 1.749; *P* = 0.047; Figure 2A2]. Tukey's *post-hoc* analysis of the freezing response over the 3-min trial showed a significant effect between control and PTSD groups only (*P* = 0.0016; Figure 2A1). While *post-hoc* analysis of freezing over 30 s blocks was shown to be significant between control and PTSD groups at the fourth and last block periods (*P* = 0.0005; *P* = 0.0003 respectively—Figure 2A2), mTBI abrogated the PTSD induced fear response to the context, and *post-hoc* analysis showed no significant effect between mTBI-PTSD and controls over the 3-min trial and also (variance) across 30 s blocks (*P* > 0.05; Figures 2A1,A2).

Two-Way ANOVA of the cued fear response for the 6-min trial session showed a significant main effect across the study groups (exposure paradigms) [*F*_(3, 90)_ = 10.47; *P* < 0.0001], and a significant main effect of time during the introduction of the auditory cue [*F*_(1, 90)_ = 47.52; *p* < 0.0001]. A main interaction was also observed between the study groups and time during exposure to the auditory cue [*F*_(3, 90)_ = 8.791; *P* < 0.0001—Figure 2B1]. A repeated measures Two-Way ANOVA conducted over 30 s blocks, over 6 min of the trial, showed a significant main effect across the study groups [*F*_(3, 45)_ = 8.190; *P* = 0.0002], and of variance across time blocks [*F*_(11, 495)_ = 36.07; *p* = 0.0001]. A main interaction was also observed between the study groups and variance across time blocks [*F*_(33, 495)_ = 6.908; *P* = 0.0001—Figure 2B2]. Tukey's *post-hoc* multiple comparisons test showed a significant effect in percent freezing time between control vs. PTSD (*P* = 0.004) and control vs. mTBI-PTSD over the 6-min trial (*P* = 0.005; Figure 2B1). This significant effect was observed following correction for multiple testing during the seventh to tenth block periods when mice were exposed to the auditory cue (*P* < 0.0001 in pair wise comparisons between control vs. PTSD and control vs. mTBI-PTSD; see Figure 2B2). Our data indicate that mTBI did not abrogate the freezing response to the cue in the mTBI-PTSD exposed animals.

For evaluation of spatial learning and memory, mice were trained for seven consecutive days prior to the induction of both mTBI and PTSD. All mice performed similarly in the pre-training seven days trial session, reaching the set criteria of one memory error (Figure 2C1). Repeated measure Two-Way ANOVA showed a significant main effect of time [*F*_(34, 952)_ = 31.41; *P* ≤ 0.0001] indicating an improvement in the rate of learning over the seven day training period. No significant effect was observed across the study groups [*F*_(3,29)_ = 2.181; *P* = 0.113].

Deficits in retrograde amnesia were assessed 1 week after the last TMT exposure and 2 weeks after the last mTBI insult, in a 5-day acquisition-training paradigm. A repeated measures Two-Way ANOVA showed no significant main effect across the study groups [*F*_(3, 28)_ = 1.357; *P* = 0.276]. A significant effect of time was observed [*F*_(24, 672)_ = 7.131; *P* < 0.001], indicating that the mice showed improvements in the rate of learning throughout the training session. No significant interaction was observed between time and study groups [*F*_(72, 672)_ = 0.942; *P* = 0.615—Figure 2C1]. Examination of the average memory errors on the last day of the training session also showed no significant effect following One-Way ANOVA [*F*_(3, 27)_ = 0.617; *P* > 0.05].

Our data therefore failed to demonstrate any significant effect of mTBI or PTSD or their combinations on spatial learning and memory performance (Figures 2C1,C2).

### Anxiety and social behaviors in a mouse model of PTSD-mTBI

**Figure 3:**
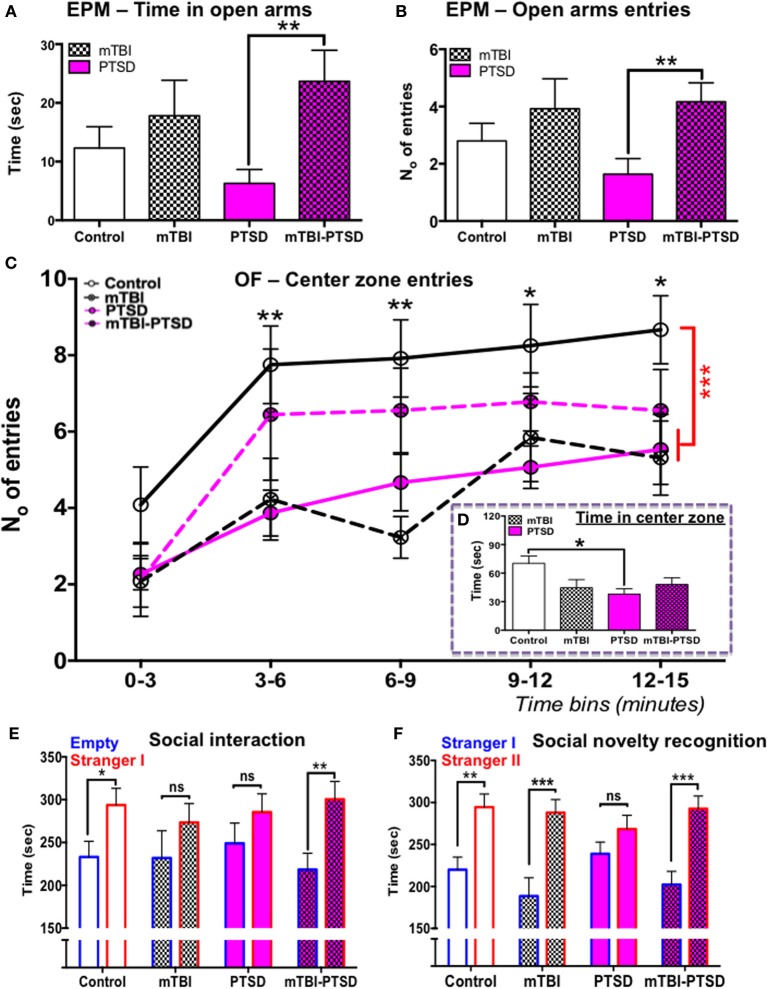
**Elevated plus maze, open-field, and social behavior**. Elevated plus maze; PTSD only group spent 50% less time on average in the open arms compared to control, however this trend was not significant (*P* > 0.05) following multiple comparisons test **(A)**. Animals that received mTBI spent relatively more time and number of entries into the open arm compared to control and PTSD only groups **(A,B)**. This comparison was shown to be statistically significant between the PTSD and mTBI-PTSD groups (*P* < 0.05). Data in **(A,B)** were analyzed using non-parametric Kruskal Wallis-One Way ANOVA followed by Dunn's correction for multiple testing. Open field test demonstrated a statistically significant reduction in mean center zone entries over the 15 min trial (*P* < 0.01) in both mTBI and PTSD animals compared to control **(C)**. However there was a noticeable trend in the behavior of mTBI-PTSD animals, compared to PTSD only and mTBI only animals. The latter showed an increased mean number of entries into the center zone, although this was not statistically significant (*P* > 0.05) compared to the other groups **(C)**. Only the PTSD group showed a significant reduction in the mean total time spent in the center zone over the 15-min trial compared to control (*P* < 0.05). These data were evident despite the fact that all groups demonstrated a comparable motor activity in the open field test (see Figure 4A). Data in **(C)** were analyzed using repeated measure-Two Way ANOVA with Tukey's *post-hoc* test, and data in **(D)** by One-Way ANOVA followed by Tukey's *post-hoc* test. Social behavior was measured in the social interaction/recognition test **(E,F)**. mTBI and PTSD only groups showed no significant distinction between the time spent in the “empty” and “stranger I” chamber **(E)**. Control and mTBI-PTSD animals performed similarly in the social interaction test **(E)**. In the social recognition test PTSD only animal group also did not show any distinction between “stranger I” and “stanger II” chambers, indicating a dysfunction in social memory and preference **(F)**. mTBI only and mTBI-PTSD animal groups performed similarly as the control group **(F)**. Data are presented as mean ± SEM. Data in **(E,F)** were analyzed using non-parametric Mann Whitney *U*-test for each group. Asterisks denote statistical significance as follows: ns; not significant; ^*^P < 0.05; ^**^P < 0.01; ^***^P < 0.001. *N* = 11–13 in all behavioral tests, except in the social interaction test (*n* = 6–8).

We investigated the effects of mTBI and PTSD on anxiety related behaviors using the elevated plus maze and the open field tests. Our observations from the elevated plus maze showed a significant main effect across the study groups (experimental paradigms) with respect to the time spent in the open arms (*P* = 0.015; Kruskal Wallis One-Way ANOVA) and entries into the open arms (*P* = 0.017; Kruskal Wallis One-Way ANOVA, see Figures 3A,B). PTSD mice spent on average 50% less time (and made 50% fewer entries) in the open arms compared to controls, however this trend was not statistically significant following multiple test correction (Dunn's) (*P* > 0.05), owing possibly to the large variation within the groups (Figures 3A,B). mTBI and mTBI-PTSD mice spent 35–50% more time (and entries) in the open arms compared to control mice and 70% more compared to PTSD only group. After correction for multiple testing, this was only significant in the combined mTBI-PTSD group compared to PTSD alone for both time (*P* = 0.007) and frequency (*P* = 0.009) of open arm entries (Figures 3A,B).

**Figure 4:**
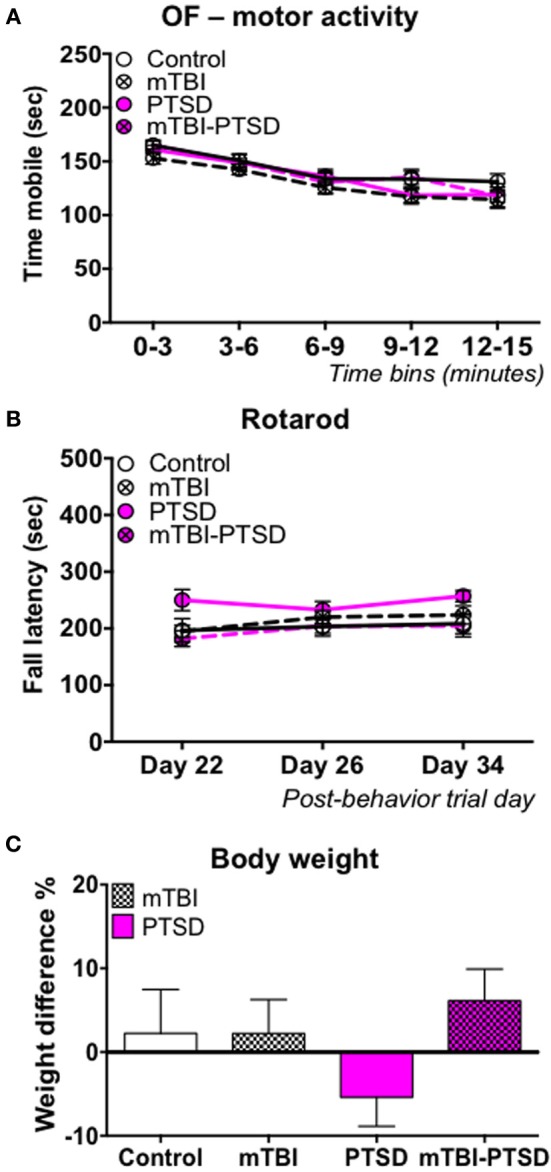
**Motor activity/coordination and growth rate**. No main effect was observed in the motor activity during the open field test, and motor coordination in the rotarod test in any of the groups **(A,B)**. Data in **(A,B)** were analyzed using repeated—Two Way ANOVA followed by *post-hoc* Tukey's test (*n* = 11–13). Mice in the control, mTBI, and combination groups showed a 2–5% increase in body weight over the 21 day stress experimental paradigm, while PTSD only mice showed a 5% reduction in their body weight **(C)**. Data are presented as mean ± SEM.

In the Open field test, analysis of center zone entries showed a significant main effect across the study groups [*F*_(3, 45)_ = 5.94; *P* = 0.002] and time [*F*_(4, 180)_ = 17.91; *P* = 0.0001], as determined by repeated measures Two-Way ANOVA (Figure 3C). No interaction was observed between the study groups and time [*F*_(12, 180)_ = 0.768; *P* = 0.683], indicating a consistency in behavior throughout the entire session across the groups, and irrespective of time. One-Way ANOVA of the center zone entries over the entire 15 min trial showed a significant main effect across the groups [*F*_(3, 45)_ = 6.32; *P* = 0.001], Tukey's *post-hoc* test showed a significant effect between control vs. PTSD (*P* = 0.004) and control vs. mTBI groups (*P* = 0.002; Figure 3C). These data indicate an increase in aversion for open space(s) in both PTSD and mTBI exposure groups compared to controls (Figure 3C). These traits were also present despite all animals having a comparable motor activity (Figure 4A). Repeated measures Two-Way ANOVA showed a significant effect on variance across the time blocks [*F*_(4, 180)_ = 49.55; *P* < 0.0001], with no effect observed across the study groups [*F*_(3, 45)_ = 1.14; *P* = 0.344], neither was there any interaction between study groups and variance across time blocks [*F*_(12, 180)_ = 0.844; *P* = 0.605].

Analyses of the open field center zone entries over time blocks showed that the combined mTBI-PTSD groups behaved distinctly different in comparison to mTBI or PTSD only groups, in that they demonstrated a consistent increase in mean number of entries into the center zone throughout the 15 min trial session (Figure 3C). Unlike the mTBI or PTSD only groups, no significant effect was observed between mTBI-PTSD vs. control following *post-hoc* Tukey's multiple comparisons test (*P* > 0.05).

With respect to time spent in the center zone, One-Way ANOVA showed a significant main effect across the groups [*F*_(3, 45)_ = 3.396; *P* = 0.026], however following *post-hoc* Tukey's test the reduction in time spent was only significant in the PTSD only group compared to controls (*P* = 0.024; Figure 3D).

Social behavior was assessed using the social interaction test and the social novelty recognition test based on the tendency of mice to respond to novel objects. Our observation shows that controls (*P* = 0.038) and, intriguingly, mTBI-PTSD (*P* = 0.002) mice demonstrated normal rodent social interaction behavior, spending significantly more time exploring the stranger mouse than the empty chamber (Figure 3E). However, the mTBI (*P* = 0.323) and PTSD (*P* = 0.387) only groups exhibited a deficit in social interaction behavior, as they failed to interact longer with the new stranger mouse than the empty chamber/cage (Figure 3E). In the social novelty test only the PTSD (*P* = 0.214) animals showed a significant deficit in their preference for the new novel stranger mouse over the old familiar mouse (Figure 3F).

### PTSD and mTBI effects on motor activity and body weight

A significant main effect of time was observed on motor activity as measured by Open field test [*F*_(4, 180)_ = 49.55; *P* < 0.0001] and, motor coordination as measured by the rotarod test [*F*_(2, 58)_ = 3.523; *P* = 0.036] following analysis by repeated measure Two-Way ANOVA (Figures 4A,B). This demonstrates a change in mobility with time during the open field test and an improvement in rotarod performance over time epochs. However, we observed no effect of either PTSD and/or mTBI on motor activity [*F*_(3, 45)_ = 1.137; *P* = 0.344], or motor coordination [*F*_(3, 29)_ = 2.42; *P* = 0.100]; all animals performed similarly, thus indicating a relatively intact motor function (Figures 4A,B). No interaction was observed between study groups and time on the motor activity measured by the open field [*F*_(12, 180)_ = 0.844; *P* = 0.605] and motor coordination measured by the rotarod test [*F*_(6, 58)_ = 1.047; *P* = 0.405]. In the PTSD only group we observed a reduction in growth rate (~5%) between the beginning and end of the 3-week stress exposure session, possibly indicating trauma-induced hypophagia, however this was not statistically significant (Figure 4C).

### Neuroendocrine and neuroimmune systemic profiles in mTBI-PTSD model

**Figure 5:**
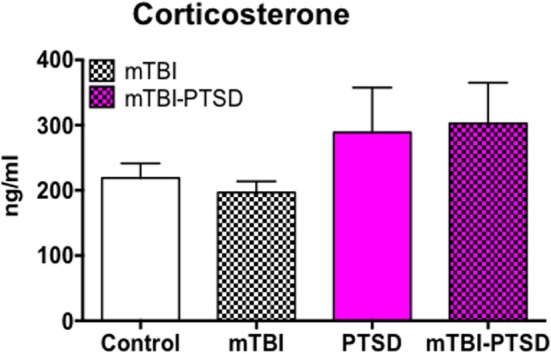
**Neuroendocrine response to mTBI and PTSD**. Plasma baseline levels of corticosterone showed a trend toward increase in PTSD and mTBI-PTSD only animals (> +70 ng/ml) compared to control. This trend did not reach statistical significance after analyses with Krukal Wallis-One Way ANOVA followed by Dunn's correction for multiple testing. mTBI animals showed a similar baseline level of corticosterone as controls. Data are presented as mean ± SEM. (*N* = 4–5).

We investigated the baseline plasma levels of the neuroendocrine stress-related hormone corticosterone and observed a trend toward an increase in plasma levels in both PTSD and PTSD-mTBI mice, however this was not statistically significant (*P* < 0.05; Kruskal Wallis One-Way ANOVA; Figure 5).

**Figure 6:**
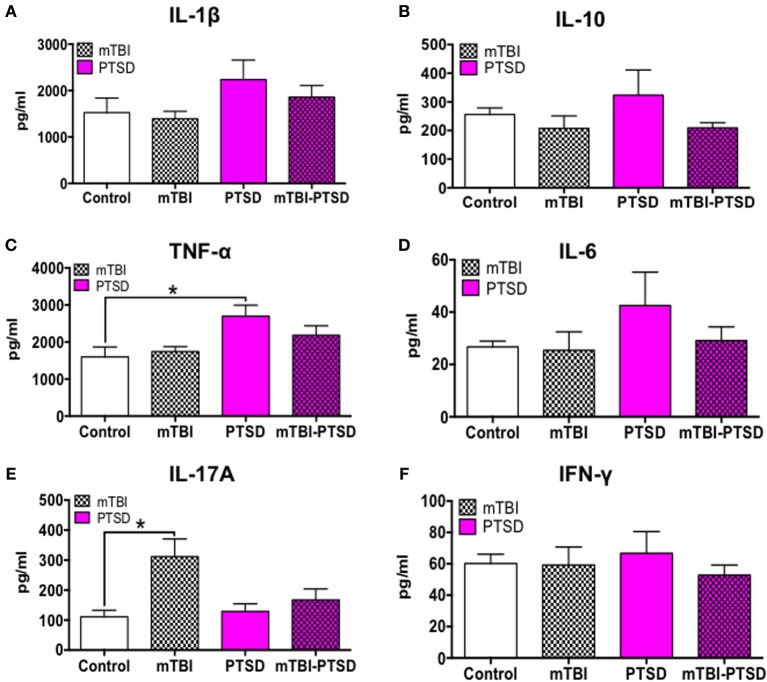
**Immune/inflammatory systemic response at acute time-point post-mTBI-PTSD**. Levels of 6 different cytokines (IL-1β, IL-10, TNFα, IL-6, IL-17A, IFNγ) were measured in the plasma to detect systemic immune/inflammatory responses to mTBI and/or PTSD. There was an obvious trend toward increase in IL-1B, IL-10, TNFα, and IL-6 in PTSD only animals compared to control and mTBI groups, however this was only statistically significant with TNFα (*P* = 0.036) and a marginal trend with IL-1β (*P* = 0.06) **(A–D)**. Intriguingly we observed a rise (*P* = 0.049) in IL-17A in mTBI only animals compared to controls **(E)**. No significant effect was seen in the levels of IFNγ amongst the different groups **(F)**. Data are presented as mean ± SEM. Data in **(A,B,D–F)** were analyzed with Kruskal Wallis-One-Way ANOVA followed multiple testing. Asterisk denote statistical significance as follows: ^*^P < 0.05. (*N* = 4–5).

Classical T-lymphocyte pro-inflammatory cytokines(s), namely Th1 (IL-1β, IL-6, TNFα, IFNγ) and Th17 (IL-17A) cytokines and the T regulatory anti-inflammatory cytokine (IL-10) were assayed using a multiplex ELISA assay kit. Our observation shows a significant main effect across the groups on TNFα plasma levels (*P* = 0.036; Kruskal Wallis One Way ANOVA), which was still evident in PTSD only mice compared to controls after correction for testing (*P* = 0.036; Figure 6C).

Consistently there was also a trend toward increase in the levels of IL-1β, IL-6, and IFNγ in PTSD only mice, however this was not statistically significant following Kruskal Wallis One-Way ANOVA (*P* > 0.05; Figures 6A,D,F).

In contrast, IL-17A plasma levels showed a significant main effect across the groups (*P* = 0.022; Kruskal Wallis One-Way ANOVA), and multiple comparisons test showed a significant increase in the Th17 cytokine (IL-17A) levels in mTBI-exposed mice compared to control (*P* = 0.016; Figure 6E). Interestingly, PTSD mitigated this rise in IL-17A levels in the PTSD only and combined mTBI-PTSD model compared to controls (*P* > 0.05). Levels of the T regulatory cytokine 1L-10 showed a trend toward an increase in the PTSD group, but were not statistically significant compared to controls (*P* > 0.05; Kruskal Wallis One-Way ANOVA; Figure 6B).

### Gross pathological changes to the brain(s) of animals exposed to PTSD and/or mTBI

Post-fixed brain hemispheres were weighed to determine any gross neuropathological outcomes; there was no observed effect of either PTSD or mTBI or both on brain weight (*P* > 0.05 Kruskal Wallis One-Way ANOVA—Table 1). We also did not observe any changes in hippocampal volume with mTBI or PTSD or both (*P* > 0.05; Kruskal Wallis One-Way ANOVA—Table 1). Consistent with our previous findings from a later timepoint (at both 6 and 12 months post-injury) in mice receiving a single mTBI (Mouzon et al., 2013), there was a notable reduction (<20%) in the volume of the corpus callosum in the mTBI and combined groups, however this was not statistically significant (*P* > 0.05 Kruskal Wallis One-Way ANOVA—Table 1).

**Table 1:** **Gross neuropathological changes in the brain**.

	**Control**	**mTBI**	**PTSD**	**mTBI-PTSD**
Brain weight (mg)	0.23 ± 0.01	0.22 ± 0.01	0.24 ± 0.003	0.23 ± 0.02
Hippocampi (mm^3^)	10.28 ± 0.57	9.41 ± 0.20	9.92 ± 0.61	9.21 ± 0.29
Corpus callosum (mm^3^)	0.92 ± 0.12	0.69 ± 0.05	0.82 ± 0.10	0.71 ± 0.08

Post-fixed brains were weighed prior to immunohistochemical and histological analyses. All groups had a relatively similar wet brain weight, no significant difference was observed between the groups (P > 0.05). Hippocampal and overlaying corpus callosum volumes (see Methods for bregma boundaries) were measured using the Cavalieri “stereology principle.” Hippocampal volume was relatively similar in all groups, no statistically significant changes were observed between the groups. There was a subtle change in the volume of the corpus callosum in mTBI and mTBI-PTSD groups compared to controls (~20% volume reduction). However this was not statistically significant following post-hoc comparisons test. Data are presented as mean ± SEM. Data (brain weight and regional volumes) were analyzed with Kruskal Wallis-One-Way ANOVA followed by Dunn's correction for multiple testing. (N = 4–5).

### Effect of mTBI and PTSD on markers of early brain injury

**Figure 7:**
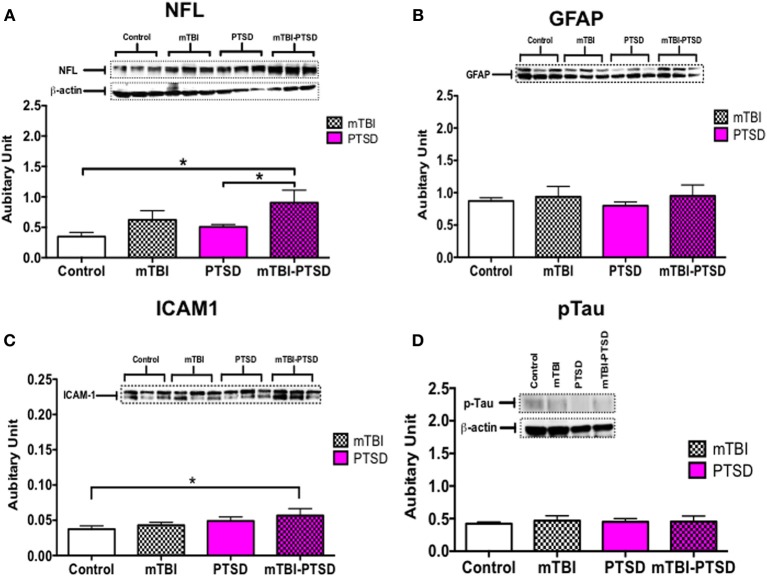
**Global brain-related changes observed at acute post-mTBI-PTSD time-point**. Hallmark potential biomarkers of mTBI (in humans) were analyzed in brain hemispheres. Western blot analysis showed a significant increase in axonal marker, neurofilament L (NFL) and inflammatory marker CD54 (ICAM)-1 levels in mTBI-PTSD groups compared to control (*P* = 0.022 and *P* = 0.016 respectively—**A,C**). No statistically significant changes were observed in astrocyte cytoskeletal protein—GFAP and phospho-tau protein (detected by s202 antibody—CP13) in all groups analyzed **(B,D)**. Data are presented as mean ± SEM. Arbitrary units were calculated by normalizing with beta-actin levels [beta-actin blot depicted in **(A)**, was used to calculate GFAP and ICAM-1 arbitrary values]. Data were analyzed with Kruskal Wallis-One-Way ANOVA followed by Dunn's correction for multiple testing. Asterisk denotes statistical significance as follows: ^*^P < 0.05. (*N* = 4–5).

Markers of axonal injury (NFL), astroglial activation (GFAP), inflammation (ICAM-1), and phospho-tau (CP13) were assessed in half brain homogenates by immunoblotting. We observed no significant change in GFAP and pTau levels globally in the brain (*P* > 0.05; Kruskal Wallis One-Way ANOVA—Figures 7B,D). Notably, axonal NFL (*P* = 0.022) and inflammatory ICAM-1 (*P* = 0.016) markers were significantly increased in the combined mTBI-PTSD groups compared to controls (Figures 7A,C). No effect was seen with mTBI or PTSD alone compared to controls, on both NFL and ICAM-1 levels (*P* > 0.05; Figures 7A,C).

### Immature neuron cell counts in the dentate gyrus following PTSD and/or mTBI

**Figure 8:**
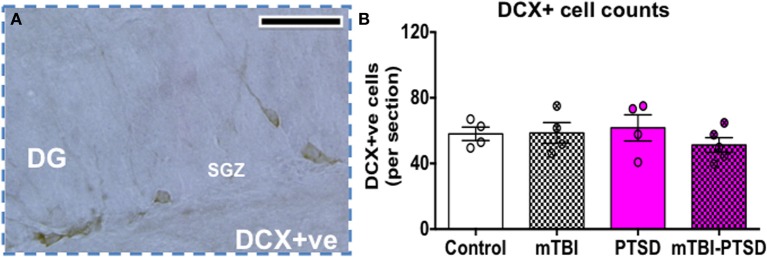
**Doublecortin (DCX) cell counts in the dentate gyrus (DG)**. Numbers of DCX+ cells were counted in the subgranular zone (SGZ) of the DG as depicted in **(A)**. No significant change was observed in the number of DCX+ cells in the subgranular zone (SGZ) of DG. Data are presented as mean ± SEM. Data were analyzed using Kruskal Wallis-One Way ANOVA followed by Dunn's correction for multiple testing. (*N* = 4–5). Scale bar represents 95 μm.

Doublecortin levels (a marker of immature neurons) were assessed in the dentate gyrus as an indicator of impaired neurogenesis/neuronal differentiation. No significant effect was seen between the groups (*P* = 0.598; Kruskal Wallis One-Way ANOVA), indicating no effect on adult neurogenesis at this acute time point with exposures to mTBI, PTSD or their combination (Figure 8).

### Astroglial and microglial cell activation

A One-Way ANOVA followed by a *post-hoc* comparisons test was performed on percent immunoreactivity area per region for cellular markers IBA1 and GFAP.

**Figure 9:**
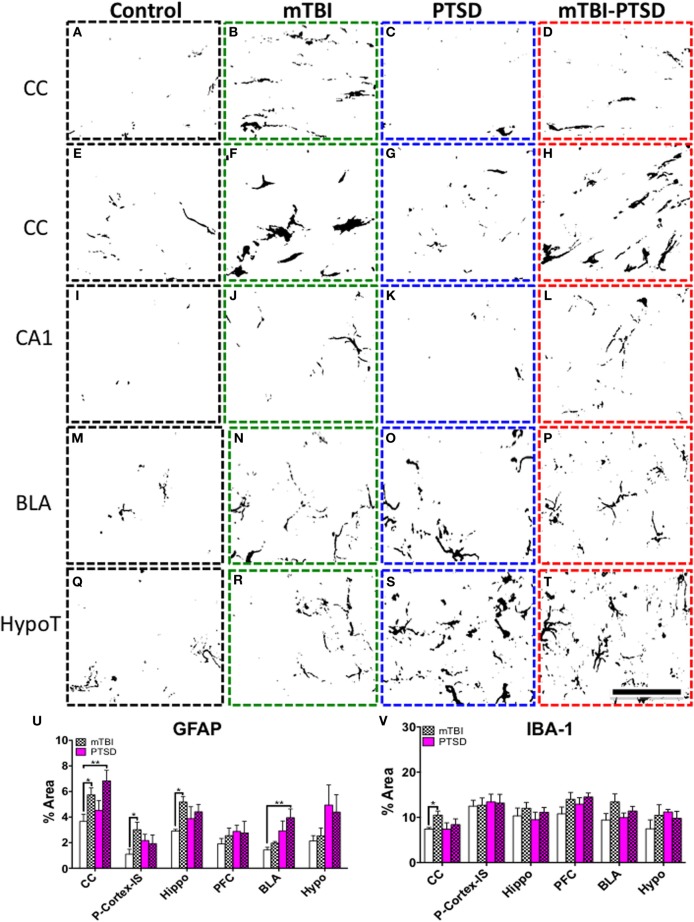
**Neuroglial response in the corpus callosum (CC), hippocampus (CA1), basolateral amygdala (BLA) and hypothalamus (HypoT), Parietal cortex- Injury site (P-Cortex-IS). (A–D)** depicts microglia IBA+ immunoreactivity in the corpus callosum (CC). There was a significant increase in mTBI animals compared to controls (*P* = 0.010); this was confirmed by quantitative densitometry (optical segmentation) **(V)**. **(E–T)** shows astrocyte GFAP+ immunoreactivity in the aforementioned brain regions. There was a significant increase in astroglial activation in the CC **(E–H)**, hippocampus **(I–L)**, and P-Cortex-IS of mTBI animals compared to controls (*P* = 0.04; *P* = 0.022, and *P* = 0.034 respectively—**U**). Likewise there was also a significant increase in astroglial activation in the CC **(E–H)**, and a marginal trend in the hippocampus **(I–L)** of mTBI-PTSD animals (*P* = 0.009; *P* = 0.09 respectively—**U**). No significant change was observed in the P-Cortex-IS of mTBI-PTSD animals compared to controls **(U)**. Notably PTSD and mTBI-PTSD animals showed an increase in GFAP immunoreactivity in the BLA **(M–P)** and HypoT by qualitative assessment **(Q–T)**. This was partly confirmed by densitometric analysis showing a significant increase in the BLA in mTBI-PTSD animals compared to controls (*P* = 0.006), and a marginal trend in PTSD mice compared to controls (*P* = 0.071). Data are presented as mean ± SEM. Data were analyzed using One-Way ANOVA followed by multiple comparisons *post-hoc* test for each brain region. A 1 in 10 series of >five average sections were analyzed per region for each cellular marker per animal. Asterisks denote statistical significance as follows: ^*^P < 0.05; ^**^P < 0.01. (*N* = 4–5). Images show segmented profiles of representative positive immunostaining. Scale bar represents 95 μm.

We observed a significant increase in IBA1 levels (*P* = 0.01) in the corpus callosum region following *post-hoc* analysis between mTBI compared to controls (Figures 9A–D,V). No significant effect was observed in the other regions (*P* > 0.05).

A robust increase in GFAP levels was also observed following *post-hoc* analysis in the corpus callosum of mTBI (*P* = 0.038) and mTBI-PTSD (*P* = 0.009) groups compared to controls (Figures 9E–H,U). mTBI also significantly increased GFAP immunoreactivity in the hippocampus (*P* = 0.022) and cortical region beneath the injury site compared to controls (*P* = 0.034; Figures 9J,L,U). These mTBI dependent effects were not consistently observed in the combined mTBI-PTSD groups compared to controls (Figure 9U). No effect was observed on GFAP immunoreactivity in the hippocampus of PTSD alone animals (Figures 9I,K,U). Increased GFAP immunereactivity was also present in the hypothalamus and basolateral amygdala of animals that received PTSD alone or combination with mTBI (Figures 9M–T,U). However, *post-hoc* analysis of the densitometry data showed a significant effect only in the mTBI-PTSD group compared to controls in the basolateral amygdala (*P* = 0.006). A marginally significant trend was observed in the PTSD only group vs. controls within the BLA (*P* = 0.071).

### Distinct neuroglial and white matter response(s) to mTBI

**Figure 10:**
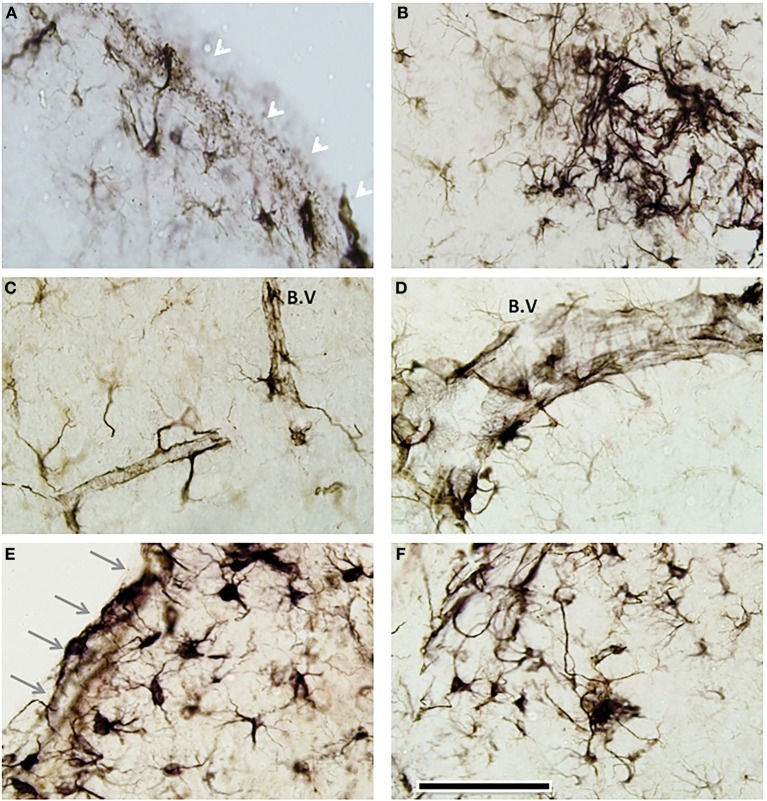
**Distinct periventricular and perivascular astroglia response to mTBI**. GFAP immunostaining revealed a layer of GFAP+ activated cells in the most superficial layer of the cortex (See white arrows in representative image—**A**), beneath the injury site of head injured animals (in both mTBI and mTBI-PTSD). There was some evidence of multifocal perivascular fibrillary astrogliosis **(B)**. In some cases these perivascular astroglial cells appeared hypertrophic with a large cell soma and thick cellular processes **(B–D)**. A distinct remarkable astrogliosis was observed in the walls of the ventricles (gray arrows—**E**) and the neighboring adjacent brain parenchyma **(E,F)**. Abbreviation; blood vessel (B.V). Scale bar represents 120 μm in all images.

We observed an increase in astroglial activation in the most superficial layer of the cortex and notably around perivascular regions of injured mice, in both mTBI and combined mTBI-PTSD groups (Figures 10A–D). These astroglial cells exhibited a hypertrophic cell somata and short thickened cellular processes (for comparison; see control resting astroglia in Figures 9A,E,I,M,Q). In the lining of the ventricles and the surrounding white matter we found a robust activation of what appeared to be hypertrophic (type-2) astroglial cells (Figures 10E,F) also demonstrating the typically large cell somata and short thick processes.

**Figure 11:**
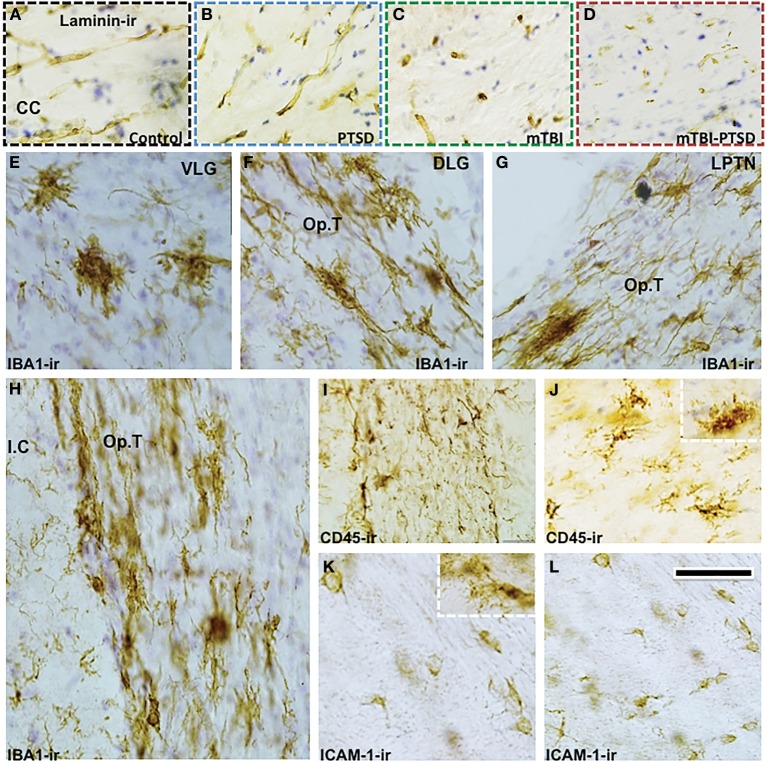
**Distinct white matter pathology and microglia response to mTBI**. There was an apparent reduction in the immunoreactivity of extracellular matrix protein—Laminin in the corpus callosum (CC) of mTBI and mTBI-PTSD animals **(A–D)**. Microglia response to injury is shown with IBA-1 and CD45 antibodies. IBA-1 staining depicted an increase in microglial activation in white matter/neighboring regions of injured animals **(E–J)**. These regions included the ventral lateral geniculate nucleus (VLG) in **(E)**, the optic tract (Op.T) projections surrounding the dorsal lateral geniculate nucleus (DLG), lateral post-thalamic nuclei (LPTN), and the internal capsule (I.C) in **(F–H)** respectively. CD45+ cell (a marker of activated microglia/macrophages) was also positive in these white matter brain regions (1, **J**). Notably ICAM-1/CD54+ cells were also observed in white matter regions of mTBI-injured animals (both mTBI and combination), they resembled oligodendroglia-like (and in some cases microglia-like cells—inset **K**) with a small round cell body and short sparse processes (see **K,L**). Scale bar in **(L)** represents 95 μm in **(A–D)**; 55 μm in **(E–G)**; 65 μm in **(H)** and 45 μm in **(I–L)**.

In the corpus callosum we noticed a reduction in the immunoreactivity levels of extracellular matrix protein marker laminin in injured mice (Figures 11A–D). Microglial cells (IBA1+ and CD45+) were also activated in other focal regions of the white matter (optic tract) and neighboring regions of injured mice (Figures 11E–J). In addition to the above histomorphological profiles, we observed ICAM1+ cells dispersed multifocally throughout some white matter regions in injured mice (Figures 11K,L), they appeared to resemble oligodendroglia-like cells (and in some cases microglial cells—see inset Figure 11K). These aforementioned histomorphological changes were comparatively similar in both mTBI and mTBI-PTSD groups; no changes were observed in the PTSD only group. PTSD did not seem to augment these changes in the combination group, therefore indicating a distinct response to mTBI.

## Discussion

### Distinct neurobehavioral patterns of mTBI-PTSD mice

At the outset of this study our goal was to develop and characterize a novel model of PTSD in conjunction with our recently developed mTBI model, in order to provide a preclinical platform with which to explore the relationship between mTBI and PTSD at the neurobehavioral, neuropathological and molecular level. With respect to the neurobehavioral outcomes, we hypothesized that an exposure to a traumatic event coupled with a concussive head injury would induce an additive significant impairment in the consolidation and/or retrieval of memory from a previously learnt task. We tested this hypothesis by training animals in a rigorous RAWM test paradigm, but 2 weeks after the last exposure to a stressful traumatic event and/or 24 days after a concussive head injury, all animals demonstrated an intact consolidation, retrieval and recall of memory from the previously learnt task in the RAWM. Previous studies have documented the effects of TBI or PTSD on memory processing in both animal and human models (Gronwall and Wrightson, 1974; Levin et al., 1976; Bremner et al., 1993; Vasterling et al., 1998; Diamond et al., 1999, 2004, 2005, 2007; Sandi and Pinelo-Nava, 2007; Mouzon et al., 2012, 2013). With respect to mTBI animal models, including the paradigm employed here, injury related cognitive deficits in both retrograde and anterograde amnesia have been documented in various permutations of cognitive tests such as Morris water maze, and the Barnes maze, from 1 week to several weeks after injury (Smith et al., 1994; Bramlett et al., 1997; Mouzon et al., 2012, 2013). However in rodent models of stress-trauma, there have been some mixed results. For example, Diamond and colleagues demonstrated that an unpredictable traumatic, stressful exposure to a predator is able to impair the consolidation and/or retrieval of memories outside the stressful environment, such as the location of a hidden platform in the water maze, 30 min–24 h post-exposure (Diamond et al., 2004, 2005, 2007), and this has been confirmed in a host of different stress-trauma animal models—(see Joels et al., 2006 for review). However when animals were tested at a later time-point (3 weeks post-stress) following exposure to two episodes of psychosocial stressors, the same authors failed to see a significant deficit in learning and memory processes in the RAWM (Zoladz et al., 2008).

In contrast to the above findings, our data suggest that stress-trauma and/or single mTBI alone or in combination do not reproduce long-lasting spatial and learning memory deficits (1–3.5 weeks respectively). Previously, in our concussive single head injury model, although we detected impairment in learning acquisition 5–7 days post-injury in the Barnes maze test, this initial deficit was resolved by 6 months post-injury (Mouzon et al., 2012, 2013). It is thus likely that any mTBI-induced deficits in learning and memory in our model were time-dependent and sensitive and, as such, due to the time-elapsed post-trauma and the mild nature of the concussive injury we were unable to detect these behavioral outcomes. In addition to this, we also consider that the highly rigorous training schedule giving to the animals prior to the stress and concussive head injury paradigm might have been counterproductive and precluded detection of some of the subtle effects in response to trauma and head injury. This behavioral pattern has been suggested by Cain et al. (1997) and demonstrated in rodents pre-trained in a Morris water maze (MWM) task. These animals were resistant to show impairment in a subsequent MWM task even after receiving a NMDAR antagonist (CGS-19775) and were able to learn the spatial location of the hidden platform at the same level as controls (see also Cain, 1997).

Despite this lack of change in the retrieval, consolidation and recall of memory from a previously learnt task in the RAWM, we report that mTBI experienced after a traumatic event significantly impairs fear memory to the spatial context but not to the discrete cue (an auditory tone). Fear/traumatic memory processing in a spatial context has been attributed primarily as a hippocampal dependent event (van der Kolk and Fisler, 1995). This hypothesis stipulates that an exposure to a traumatic event interferes with the hippocampal based memory processing resulting in the dissociation and fragmented storage of memory imprinted as affective and perceptual states that remain unmodified for many months or years (van der Kolk and Fisler, 1995). We have previously shown that a single mTBI can alter hippocampal integrity (notably alteration to glial cells) 10 days post-injury (Mouzon et al., 2012), and thus possibly the subsequent disruption of fragmented processing of the traumatic memory response to a contextual spatial stimulus as shown herein. This indicates that the combination of a traumatic event and a concussive head injury can preclude some of the fear or anxiety-related responses to the contextual environment in which both occurred. The apparent lack of head injury effect on the Cued fear memory response is also informative, and perhaps supports the theory that certain common traits associated with PTSD (such as a precipitation of anxiety in response to a powerful environmental cue associated with the traumatic event) are not mutually exclusive when it occurs with a mTBI. Fear memory in response to a potentially life threatening stimulus has been suggested to involve the formation and storage of memory primarily in the amygdala, as a result of an intense and rapid activation of endogenous plasticity (van der Kolk and Fisler, 1995; Rauch et al., 2006; Diamond et al., 2007; Shin and Liberzon, 2010). The amygdala is imbedded deep in the medial temporal lobe structures and, as such, is more protected from the traumatic head injury inflicted to the top of the parietal cortex in our model. This implies that the enhanced processing in the amygdala in response to a traumatic cue is not significantly influenced by the mTBI or loss of consciousness in our model and, as such, mTBI does not fully guarantee protection from trauma-related intrusive memories or PTSD symptoms. Our data demonstrating the differential effect of mTBI on both Contextual and discrete Cued fear memory responses are in agreement with the human study literature showing inconclusive and sometimes conflicting results on the prevalence of PTSD in some mTBI cases; in particular, this agrees with the observation that some of these patients only elicit partial (and fewer) intrusive clusters of PTSD symptoms that do not fully meet the criteria for the disorder (Ohry et al., 1996; Warden et al., 1997; Gil et al., 2005; Bryant, 2008). Three other animal models involving a single lateral fluid percussion injury, repetitive (×2) closed head injury and repetitive (×3) blast over-pressure injury have investigated the influence of mTBI on fear conditioning (Witgen et al., 2005; Elder et al., 2012; Klemenhagen et al., 2013). Both repetitive closed head and blast injuries failed to show any significant effect on contextual fear response, while a single lateral fluid percussion injury significantly inhibited the Contextual fear memory response seen in non-injured animals. In the Cued fear memory test, the blast injury model demonstrated an increase in freezing behavior compared to the un-injured groups, while the closed head injury model did not reveal any changes in freezing behaviors to the discrete Cue. Although these findings show differing and conflicting results, it is likely that subtle differences in the experimental paradigm (such as nature of injury, blast vs. concussion; frequency of injury; single vs. repetitive) and timeline of procedures (such as time and separation of injury in relation to conditioning experiments; pre vs. post; days vs. weeks) might have all factored and contributed to the inconsistent behavioral outcomes. Furthermore this also highly suggests that mTBI can influence different aspects associated with presentation of PTSD, and thus indicates an overlapping neurobiological mechanism that can be inhibited and/or exacerbated depending on the nature of the TBI.

In addition we observed other distinct behavioral traits in our model. Animals in the PTSD groups, as expected, exhibited increased anxiety like behavior, impairment in social behavior and hypophagia. mTBI and mTBI-PTSD groups also exhibited some evidence of anxiety-like behavior in the open field test, but this was also coupled with an increase in disinhibitory like behavioral trends in the elevated plus maze and social novelty recognition test (and this appeared to be ostensibly increased in the combination groups). Notably, although mTBI animals performed normally in social novelty recognition tests, they exhibited an impaired social interaction, indicating a reduced motivation in these mice to affiliate with a novel conspecific. Reasons behind these data are unknown, but further demonstrate the clinical complexity in human TBI cases and the heterogeneity of the condition (Bogdanova and Verfaellie, 2012). These data suggest that mTBI is likely related to a composite of behavioral (disinhibitory) traits typified by impulsivity, poor risk assessment and indecisiveness and further implicate possible diffuse structural damage to the frontal lobe and/or altered neurotransmitter systems (such as dopaminergic or serotonergic pathways) involved in the control of such behavioral traits.

### Systemic changes in mTBI-PTSD model

After observing the different behavioral traits in our model, we examined some of the physiological changes that might be responsible for these responses to both trauma and head injury. We examined plasma levels of corticosterone, and observed a trend toward an increase in PTSD groups, possibly indicating an exaggerated adrenal response. Empirical work from the literature has documented mixed results in relation to neuroendocrine profiles and hypothalamic-pituitary-adrenal (HPA) axis function in PTSD patients and animal models, reporting abnormally low baseline cortisol/corticosterone levels compared to control subjects (Mason et al., 1986; Yehuda et al., 1995a; Goenjian et al., 1996; Seedat et al., 2003; Rohleder et al., 2004; Zoladz et al., 2012), while others have reported an elevation in their levels (Kant et al., 1987; Pitman and Orr, 1990; Maes et al., 1998; Bremner et al., 2003; Young and Breslau, 2004; Kwon et al., 2011; see Zoladz and Diamond, 2013 for further extensive review). Others have also reported an increase in sensitivity and density of glucocorticoid receptors, adrenal hypertrophy, and an increased dexamethasone induced suppression of cortisol and adrenocorticotrophine hormone (ACTH), indicating an enhanced negative feedback (Yehuda et al., 1995b; Goenjian et al., 1996; Stein et al., 1997; Duval et al., 2004). Reasons behind these heterogeneous findings in the literature, although unknown, may be uniquely related to distinct types or nature of traumatic events (acute vs. chronic, single vs. repeated, psychosocial vs. physical trauma) or species differences.

Although human studies suggest severity-dependent alterations in HPA axis function post-TBI (Yuan and Wade, 1991; Cohan et al., 2005; Tanriverdi et al., 2010; Baxter et al., 2013), in our mouse study mTBI alone did not alter corticosterone levels compared to controls. Moreover, the combination of stress and injury also did not abrogate or augment levels of corticosterone, compared to stress only groups.

In human studies, levels of inflammatory cytokines (IL-6, TNFα, 1L-1β) have been correlated to the severity of mTBI consequences and PTSD in some patients (Spivak et al., 1997; Baker et al., 2001; Gola et al., 2013; Woodcock and Morganti-Kossmann, 2013). We probed plasma samples for different cytokines and observed a significant increase in TNFα in stress only exposed animals, and also a trend toward increase in IL-6 and IL-1β. A inflammatory state typified by alteration in both humoral (pro/anti-inflammatory cytokines) and cellular factors (immune cells; CD4+ T cells, and macrophages) from the innate and adaptive immune system has been suggested to be associated with depression-anxiety like behaviors in humans and animal models (Dantzer et al., 2008; Dowlati et al., 2010; Kwon et al., 2011). The causative factor leading to an increase in low-grade proinflammatory response to trauma is unknown, but could possibly be attributed to an exaggerated sympathetic nervous system activity and the release of adrenal hormones/neurotransmitters such as norepinephrine, epinephrine, or dopamine that can both directly and indirectly activate immune cell responses. This apparent increase in pro-inflammatory cytokines can be detrimental to neurobiological systems involved in the regulation of stress-anxiety behaviors. For example, they can affect neurotransmitter systems by stimulating re-uptake of monoamines from the synapse through the increase in the activity and density of transporters of stress-related neurotransmitters: norepinephrine, dopamine, and 5HT (Zoladz and Diamond, 2013). Further evidence also suggests that proinflammatory cytokines may disrupt the capacity of stress related glucocorticoid receptors to translocate to the nucleus where they exert their action to suppress the activity of pro-inflammatory transcription factors such as nuclear factor kappa B (NF-kB) (Dantzer et al., 2008). Our data therefore seems to support a classical T-lymphocyte (Th1) proinflammatory cell response in possibly driving the pathogenesis and pathophysiology of PTSD in our model. In addition to the changes in proinflammatory cytokines we also observed a trend towards increase in the levels of anti-inflammatory cytokine 1L-10. A neuroprotective role for IL-10 in preventing depression-anxiety like behavior has been postulated. Rodents with IL-10 over expression display less anxiety-like behavior, while IL-10 knockouts display a greater depression-like behavior in a forced swim test (Mesquita et al., 2008). Moreover, administering 1L-10 attenuated the symptoms associated with a model of sickness behavior, a syndrome which shares many common features with major depression, including listlessness, anhedonia, hypophagia, and social withdrawal (Bluthe et al., 1999). These data are noteworthy as they possibly suggest an attempted protective T regulated cell immunosurveillance mechanism by the body to the trauma induced physiological changes.

Intriguingly in animals exposed only to mTBI, a significant increase (×3 fold) in the Th17 cytokine IL-17A was observed compared to controls, and this effect was abrogated in the combination groups, possibly indicating separate systemic inflammatory response(s) to PTSD and head injury, with mTBI involving a Th17 classical T lymphocyte inflammatory response at this acute time point in our model. In interpreting these data it is noteworthy to consider that these systemic changes only reflect a snapshot view at a single time-point post-stress/injury and, as such, it cannot be excluded that other cytokine profile responses could have occurred prior to the changes observed 2 weeks post-exposure and 24 days post-concussion. Nonetheless our data at least seem to implicate an early robust systemic T-lymphocyte (Th17 cell) pro-inflammatory response to concussive head injury.

In mice receiving PTSD and mTBI exposures we failed to observe any distinct trend in cytokine profiles. Although this result is puzzling it further adds to the complexity of the interaction between mTBI and its influence on PTSD related mechanisms, that can make the diagnosis, management and treatment of both conditions individually or in their combination very challenging (see Vasterling et al., 2009; Zoladz and Diamond, 2013 for further discussion).

### Brain specific changes in the mTBI-PTSD model

#### Gross neuropathological changes in a model of mTBI and PTSD individually or in combination

We assessed brain tissue of animals exposed to stress and concussion for any gross neuropathological outcomes. Our data did not reveal any gross anatomical changes to brain weight, nor hippocampal volume, which has been ostensibly associated with the development and/or predisposition to PTSD in humans and animal models (Zoladz and Diamond, 2013). In line with our previous study we also observed a slight reduction in white matter (corpus callosum) volume in head injured mice (Mouzon et al., 2012, 2013). The primary hallmark pathological feature of mTBI is diffuse axonal injury, which occurs in the absence of any gross anatomical changes that can be visibly imaged with a conventional CT or MRI scan. This damage to the integrity of the white matter is caused by the initial shearing and tensile forces from the impact to the head and is well documented in humans (see seminal work by Rand and Courville, 1946 and review papers Povlishock, 1993 and Johnson et al., 2013) and thus further supports the relevance of our concussive head injury model with the human scenario.

#### Effects of trauma on acute biomarkers of brain injury

Several biomarkers of mTBI have already been established, these include proteins that indicate axonal injury, astroglial damage, BBB dysfunction and neuroinflammation (Dekosky et al., 2013; Zetterberg et al., 2013). We examined the effects that a background of repeated stress-trauma may have on selective markers of early brain injury (GFAP, NFL, ICAM-1, pTau). Initially we did not expect to see any global changes in these markers from brain homogenates examined 24 days after injury, due to the mild nature of the single injury and the time elapsed post-injury. We were therefore intrigued to discover that there was a prolonged persistent increase in axonal injury and inflammatory markers—NFL and ICAM-1 respectively in our combination group. Stress-trauma exposure on its own did not affect the levels of these biomarkers. These results suggests that individuals exposed to a mTBI in combination with repeated life-threatening traumatic situations (as regularly encountered by combat soldiers) may exhibit a greater magnitude of neurological damage in the long term due partly to a slower neuro-reparative mechanism, compared to head injured veterans who are not exposed to repeated traumatic events.

#### Regional changes in neurogenesis and neuroglial activation

As mentioned above the neurocircuitry models of PTSD implicate frontal and limbic (amygdala and hippocampus) structures as neurobiological substrates responsible for fear response and formation of fear associations. A large body of evidence supports an exaggerated responsivity of the amygdala with the concurrent dampening of the (inhibitory) prefrontal cortex and hippocampal activity (van der Kolk and Fisler, 1995; Rauch et al., 2006; Diamond et al., 2007; Vasterling et al., 2009; Shin and Liberzon, 2010). In mTBI cases, studies have demonstrated a vulnerability of the hippocampus, frontal white matter, and subcortical structures with white matter projections to the frontal cortex (Povlishock, 1993). It is currently unclear what direct effects mTBI has on these frontal and limbic structures in their processing of learned fear responses and formation of fear associations at the cellular level.

We investigated the effect that mTBI or PTSD either individually or in combination with each other might have on aspects of neurogenesis (doublecortin) and neuroglial activation (IBA1, GFAP) in these aforementioned brain regions.

Although previous animal models have suggested a reduction in neurogenesis post-trauma (Dranovsky and Hen, 2006) and an induction in neuronal proliferation post-TBI (Kwon et al., 2011; Gao and Chen, 2013), our investigation of the immature neuronal marker doublecortin in the DG, a hippocampal subfield where postnatal neurogenesis is known to occur, revealed no significant effect of PTSD, mTBI or their combination. This finding is in accordance with a most recent study, which investigated the acute effects of psychosocial stressors in combination with a moderate controlled cortical impact injury, and reported no effect or interaction between PTSD and mTBI on DCX levels in the subgranular zone (SGZ) (Acosta et al., 2013).

Neuroglial activation features prominently in CNS neurotrauma, and is a hallmark feature of our mTBI model (Mouzon et al., 2013). We investigated microglial (IBA1+) and astroglial (GFAP+) activation in our models in different brain regions related to both PTSD and mTBI. We did not observe any significant changes in microglial activation in PTSD animals in any of the brain regions examined. As previously reported there was a localized increase in microglial activation in the white matter of mTBI animals (Mouzon et al., 2012, 2013), however, this was abrogated in the combination groups, suggesting a differential microglial response to injury in the context of trauma. Whether these effects are protective or time-dependent in nature remains to be determined. From our systemic inflammatory cytokine data, we observed differential responses in the mTBI (increased IL-17A) and PTSD (increased TNFα) groups. We were unable to measure regional brain cytokine changes, and thus are uncertain whether these systemic changes might impact on local brain inflammatory responses. Giving the relative lack of widespread microglial activation in the brain in general, we consider that the systemic cytokine responses are of a mild nature or downstream of other mechanisms, and possibly do not impact on central inflammatory processes.

Changes to astroglial cells were more pronounced compared to microglial cells, and were observed in localized regions. An apparent increase in GFAP immunoreactivity was observed in the BLA and hypothalamus of PTSD and combination groups, and this was absent in the mTBI only group, indicating a localized PTSD-dependent astroglial response to trauma in these regions notably involved in control of stress mechanisms.

Given that we also observed a slightly greater increase in GFAP levels within the BLA in the combination groups compared to PTSD alone, indirect influences of the impact of injury in the BLA cannot be completely excluded.

Specifically our analyses of the hypothalamus consisted of the combined medial and periventricular zones. These regions contain cytoarchitectonically distinct subnuclei regions such as the paraventricular, dorsal-medial, ventromedial nuclei, that play a role in neurohypophysis (release of hormones), regulating neuroendocrine and autonomic functions. Although we were unable to conduct our analyses for each distinct subnuclei separately, our data still suggest significant widespread changes within these nuclear groups, and a likely impairment in hypothalamic regulations of overarching functions that pertain to stress-related neuroendocrine functions.

The basis of the differences in microglial vs. astroglial responses in the brain regions we have investigated are unclear, one possibility is that the response of microglia in these regions may develop at different rates or endure for different periods and, as such, could have resolved prior to the timepoint examined herein.

In addition to the white matter pathologies, we also report a distinct pathological feature of our mTBI model not previously documented, typified by a reduction in vascular extracellular matrix protein (laminin), an increase in ICAM1+ oligodendroglia like cells in the white matter, and a localized perivascular and periventricular activation of microglial and astroglial cells. The latter reminiscent of CTE like pathology in humans (Omalu et al., 2011).

## Conclusion

A major issue in the diagnosis of PTSD and mTBI in their comorbid form is the clinical heterogeneity and the co-occurrence of overlapping clinical symptoms such as emotional numbing, insomnia, fatigue, depression, anxiety, and amnesia. Despite the numerous epidemiological studies conducted thus far highlighting the cluster of PTSD symptoms in patients exposed to a mild/moderate head injury, the consequences and the mechanism(s) of interaction between both disorders remain elusive. This is partly due to the methodological restraints inherent in human studies, such as the difficulties associated with recruiting sufficient numbers of unbiased samples, and conducting longitudinal and prospective study designs. At the outset of this study we set out to combine our already established concussive head injury mouse model, which has been extensively characterized from 24 h to 24 months post-injury (Mouzon et al., 2012, 2013), with a newly developed model of PTSD. The PTSD model was specifically tailored to capture the critical aspects of PTSD symptomatology prevalent in combat veterans, with face and constructive validity.

We have utilized an ethologically relevant model of unpredictable inescapable predator stress exposure and physical trauma (footshock) to replicate a debilitating feeling of intense fear, horror, and helplessness as experienced by PTSD patients (Criterion A of DSM-5). The face validity of our model was determined by post-exposure behavioral phenotypes that were analogous to the human condition as defined by the first four criteria's of the DSM-5. (i) PTSD animals showed evidence of intrusive traumatic memories (criterion B of DSM-5) in the cued and contextual fear conditioning experiments. (ii) Anxiety and avoidance like behavior were observed in the elevated plus maze and open field-tests (Criterion C of DSM-5). (iii) Negative alterations in social behavior were demonstrated by the impairment in social interaction and novelty recognition tests (criterion D of DSM-5). With respect to the constructive validation of our model, post-stress neurobiological assessment demonstrated evidence of systemic inflammation and a trend toward neuroendocrine alterations.

Other aspects of our model that require further investigation includes: the sustained long-term expression of post-exposure phenotypes (>1–3 months), the predictive and discriminative validity of our model, to assess outcomes of novel pharmacotherapy, and inherent variability seen in PTSD patients. For future optimization of our model, because we have focused on a repeated TMT exposure paradigm we consider it important in further development to also include a fear conditioning/extinction measure for the repeated TMT stressors. This will enable assessment of intrusive traumatic memories to the predator odor when exposed to a reminder cue or a novel context. Given the importance of psychosocial factors in the development of PTSD in combat veterans, we also plan to consider including a paradigm that will involve social instability to improve our model (see Zoladz et al., 2008, 2012). To optimize our cognitive paradigm we will also eliminate the pre-training session and include post-exposure tests with both the RAWM and the Barnes maze tests.

In addition to the PTSD related phenotypes, we have also observed that when animals are exposed to both trauma and mTBI, they exhibit overlapping and distinct neurobehavioral traits, involving abnormalities in anxiety and social behavior. This complexity in behavioral phenotypes is also evident in the systemic and brain specific changes that occur in these animals, typified by differences in (i) systemic immune response, (ii) brain inflammatory and (iii) axonal markers of injury, (iv) astroglial activation and (v) plasticity. These studies have serious implications when considering the management and diagnosis of patients suffering from either PTSD/mTBI or both (comorbid) conditions. We propose that this model is a useful tool to assess some of these vital interactions between both psychological and biomechanical trauma and for the identification of novel biomarkers and therapeutic strategies to ameliorate the neurological consequences. Our future studies will focus on characterizing our model in the long-term at extended time points post-exposure (months to years), and also involve applying omic technologies to identify the molecular pathways implicated in the processes underlying co-morbid mTBI-PTSD.

## Statement of VA support and opinion

David M. Diamond and Fiona Crawford were supported by Career Scientist and/or Merit Review Awards from the Veterans Affairs Department during the production of this review. The opinions expressed in this review are those of the authors and not of the Department of Veterans Affairs or the US government.

### Conflict of interest statement

The authors declare that the research was conducted in the absence of any commercial or financial relationships that could be construed as a potential conflict of interest.

## References

[B1] Acosta S. A. Tajiri N. Shinozuka K. Ishikawa H. Grimmig B. Diamond D. (2013). Long-term upregulation of inflammation and suppression of cell proliferation in the brain of adult rats exposed to traumatic brain injury using the controlled cortical impact model. PLoS ONE 8:e53376 10.1371/journal.pone.0053376 23301065 PMC3536766

[B2] American Psychiatric Association. (2013). Diagnostic and Statistical Manual of Mental Disorders, 5th Edn. Arlington, VA: American Psychiatric Publishing

[B3] Baker D. G. Ekhator N. N. Kasckow J. W. Hill K. K. Zoumakis E. Dashevsky B. A. (2001). Plasma and cerebrospinal fluid interleukin-6 concentrations in posttraumatic stress disorder. Neuroimmunomodulation 9, 209–217 10.1159/000049028 11847483

[B4] Barnes S. M. Walter K. H. Chard K. M. (2012). Does a history of mild traumatic brain injury increase suicide risk in veterans with PTSD? Rehabil. Psychol. 57, 18–26 10.1037/a0027007 22369114

[B5] Baxter D. Sharp D. J. Feeney C. Papadopoulou D. Ham T. E. Jilka S. (2013). Pituitary dysfunction after blast traumatic brain injury: the UK BIOSAP study. Ann. Neurol. 74, 527–536 10.1002/ana.23958 23794460 PMC4223931

[B6] Bazarian J. J. Donnelly K. Peterson D. R. Warner G. C. Zhu T. Zhong J. (2013). The relation between posttraumatic stress disorder and mild traumatic brain injury acquired during operations enduring freedom and Iraqi freedom. J. Head Trauma Rehabil. 28, 1–12 10.1097/HTR.0b013e318256d3d3 22647965

[B7] Bluthe R. M. Castanon N. Pousset F. Bristow A. Ball C. Lestage J. (1999). Central injection of IL-10 antagonizes the behavioural effects of lipopolysaccharide in rats. Psychoneuroendocrinology 24, 301–311 10.1016/S0306-4530(98)00077-8 10101735

[B8] Bogdanova Y. Verfaellie M. (2012). Cognitive sequelae of blast-induced traumatic brain injury: recovery and rehabilitation. Neuropsychol. Rev. 22, 4–20 10.1007/s11065-012-9192-3 22350691 PMC4372457

[B9] Bramlett H. M. Green E. J. Dietrich W. D. (1997). Hippocampally dependent and independent chronic spatial navigational deficits following parasagittal fluid percussion brain injury in the rat. Brain Res. 762, 195–202 10.1016/S0006-8993(97)00387-9 9262173

[B10] Bremner J. D. Scott T. M. Delaney R. C. Southwick S. M. Mason J. W. Johnson D. R. (1993). Deficits in short-term memory in posttraumatic stress disorder. Am. J. Psychiatry 150, 1015–1019 8317569 10.1176/ajp.150.7.1015

[B11] Bremner J. D. Vythilingam M. Vermetten E. Adil J. Khan S. Nazeer A. (2003). Cortisol response to a cognitive stress challenge in posttraumatic stress disorder (PTSD) related to childhood abuse. Psychoneuroendocrinology 28, 733–750 10.1016/S0306-4530(02)00067-7 12812861

[B12] Bryant R. A. (2008). Disentangling mild traumatic brain injury and stress reactions. N. Engl. J. Med. 358, 525–527 10.1056/NEJMe078235 18234757

[B13] Cain D. P. (1997). LTP, NMDA, genes and learning. Curr. Opin. Neurobiol. 7, 235–242 10.1016/S0959-4388(97)80012-8 9142751

[B14] Cain D. P. Saucier D. Boon F. (1997). Testing hypotheses of spatial learning: the role of NMDA receptors and NMDA-mediated long-term potentiation. Behav. Brain Res. 84, 179–193 10.1016/S0166-4328(96)00149-0 9079784

[B15] Cohan P. Wang C. McArthur D. L. Cook S. W. Dusick J. R. Armin B. (2005). Acute secondary adrenal insufficiency after traumatic brain injury: a prospective study. Crit. Care Med. 33, 2358–2366 10.1097/01.CCM.0000181735.51183.A7 16215393

[B16] Dantzer R. O'Connor J. C. Freund G. G. Johnson R. W. Kelley K. W. (2008). From inflammation to sickness and depression: when the immune system subjugates the brain. Nat. Rev. Neurosci. 9, 46–56 10.1038/nrn2297 18073775 PMC2919277

[B17] Daskalakis N. P. Yehuda R. Diamond D. M. (2013). Animal models in translational studies of PTSD. Psychoneuroendocrinology 38, 1895–1911 10.1016/j.psyneuen.2013.06.006 23845512

[B18] Dekosky S. T. Blennow K. Ikonomovic M. D. Gandy S. (2013). Acute and chronic traumatic encephalopathies: pathogenesis and biomarkers. Nat. Rev. Neurol. 9, 192–200 10.1038/nrneurol.2013.36 23558985 PMC4006940

[B19] Diamond D. M. Campbell A. M. Park C. R. Halonen J. Zoladz P. R. (2007). The temporal dynamics model of emotional memory processing: a synthesis on the neurobiological basis of stress-induced amnesia, flashbulb and traumatic memories, and the Yerkes-Dodson law. Neural Plast. 2007:60803 10.1155/2007/60803 17641736 PMC1906714

[B20] Diamond D. M. Park C. R. Campbell A. M. Woodson J. C. (2005). Competitive interactions between endogenous LTD and LTP in the hippocampus underlie the storage of emotional memories and stress-induced amnesia. Hippocampus 15, 1006–1025 10.1002/hipo.20107 16086429

[B21] Diamond D. M. Park C. R. Heman K. L. Rose G. M. (1999). Exposing rats to a predator impairs spatial working memory in the radial arm water maze. Hippocampus 9, 542–552 10560925 10.1002/(SICI)1098-1063(1999)9:5<542::AID-HIPO8>3.0.CO;2-N

[B22] Diamond D. M. Park C. R. Woodson J. C. (2004). Stress generates emotional memories and retrograde amnesia by inducing an endogenous form of hippocampal LTP. Hippocampus 14, 281–291 10.1002/hipo.10186 15132427

[B23] Dowlati Y. Herrmann N. Swardfager W. Liu H. Sham L. Reim E. K. (2010). A meta-analysis of cytokines in major depression. Biol. Psychiatry 67, 446–457 10.1016/j.biopsych.2009.09.033 20015486

[B24] Dranovsky A. Hen R. (2006). Hippocampal neurogenesis: regulation by stress and antidepressants. Biol. Psychiatry 59, 1136–1143 10.1016/j.biopsych.2006.03.082 16797263 PMC7537828

[B25] Duval F. Crocq M. A. Guillon M. S. Mokrani M. C. Monreal J. Bailey P. (2004). Increased adrenocorticotropin suppression after dexamethasone administration in sexually abused adolescents with posttraumatic stress disorder. Ann. N.Y. Acad. Sci. 1032, 273–275 10.1196/annals.1314.036 15677426

[B26] Elder G. A. Dorr N. P. De Gasperi R. Gama Sosa M. A. Shaughness M. C. Maudlin-Jeronimo E. (2012). Blast exposure induces post-traumatic stress disorder-related traits in a rat model of mild traumatic brain injury. J. Neurotrauma 29, 2564–2575 10.1089/neu.2012.2510 22780833 PMC3495123

[B27] Gao X. Chen J. (2013). Moderate traumatic brain injury promotes neural precursor proliferation without increasing neurogenesis in the adult hippocampus. Exp. Neurol. 239, 38–48 10.1016/j.expneurol.2012.09.012 23022454 PMC3755608

[B28] Gil S. Caspi Y. Ben-Ari I. Z. Koren D. Klein E. (2005). Does memory of a traumatic event increase the risk for posttraumatic stress disorder in patients with traumatic brain injury? A prospective study. Am. J. Psychiatry 162, 963–969 10.1176/appi.ajp.162.5.963 15863799

[B29] Goenjian A. K. Yehuda R. Pynoos R. S. Steinberg A. M. Tashjian M. Yang R. K. (1996). Basal cortisol, dexamethasone suppression of cortisol, and MHPG in adolescents after the 1988 earthquake in Armenia. Am. J. Psychiatry 153, 929–934 8659616 10.1176/ajp.153.7.929

[B30] Gola H. Engler H. Sommershof A. Adenauer H. Kolassa S. Schedlowski M. (2013). Posttraumatic stress disorder is associated with an enhanced spontaneous production of pro-inflammatory cytokines by peripheral blood mononuclear cells. BMC Psychiatry 13:40 10.1186/1471-244X-13-40 23360282 PMC3574862

[B31] Gronwall D. Wrightson P. (1974). Delayed recovery of intellectual function after minor head injury. Lancet 2, 605–609 10.1016/S0140-6736(74)91939-4 4137603

[B32] Hoge C. W. McGurk D. Thomas J. L. Cox A. L. Engel C. C. Castro C. A. (2008). Mild traumatic brain injury in U.S. soldiers returning from Iraq. N. Engl. J. Med. 358, 453–463 10.1056/NEJMoa072972 18234750

[B33] Joels M. Pu Z. Wiegert O. Oitzl M. S. Krugers H. J. (2006). Learning under stress: how does it work? Trends Cogn. Sci. 10, 152–158 10.1016/j.tics.2006.02.002 16513410

[B34] Johnson V. E. Stewart. W. Smith D. H. (2013). Axonal pathology in traumatic brain injury. Exp. Neurol. 248, 35–43 10.1016/j.expneurol.2012.01.013 22285252 PMC3979341

[B35] Kant G. J. Leu J. R. Anderson S. M. Mougey E. H. (1987). Effects of chronic stress on plasma corticosterone, ACTH and prolactin. Physiol. Behav. 40, 775–779 10.1016/0031-9384(87)90282-4 2823307

[B36] Klemenhagen K. C. O'Brien S. P. Brody D. L. (2013). Repetitive concussive traumatic brain injury interacts with post-injury foot shock stress to worsen social and depression-like behavior in mice. PLoS ONE 8:e74510 10.1371/journal.pone.0074510 24058581 PMC3776826

[B37] Kwon S. K. Kovesdi E. Gyorgy A. B. Wingo D. Kamnaksh A. Walker J. (2011). Stress and traumatic brain injury: a behavioral, proteomics, and histological study. Front. Neurol. 2:12 10.3389/fneur.2011.00012 21441982 PMC3057553

[B38] Levin H. S. Grossman R. G. Kelly P. J. (1976). Aphasic disorder in patients with closed head injury. J. Neurol. Neurosurg. Psychiatry 39, 1062–1070 10.1136/jnnp.39.11.1062 1011017 PMC1083304

[B39] Maes M. Lin A. Bonaccorso S. Van Hunsel F. Van Gastel A. Delmeire L. (1998). Increased 24-hour urinary cortisol excretion in patients with post-traumatic stress disorder and patients with major depression, but not in patients with fibromyalgia. Acta Psychiatr. Scand. 98, 328–335 10.1111/j.1600-0447.1998.tb10092.x 9821456

[B40] Mason J. W. Giller E. L. Kosten T. R. Ostroff R. B. Podd L. (1986). Urinary free-cortisol levels in posttraumatic stress disorder patients. J. Nerv. Ment. Dis. 174, 145–149 10.1097/00005053-198603000-00003 3950596

[B41] McAllister T. W. Stein M. B. (2010). Effects of psychological and biomechanical trauma on brain and behavior. Ann. N.Y. Acad. Sci. 1208, 46–57 10.1111/j.1749-6632.2010.05720.x 20955325 PMC3169086

[B42] McFall M. E. Veith R. C. Murburg M. M. (1992). Basal sympathoadrenal function in posttraumatic distress disorder. Biol. Psychiatry 31, 1050–1056 10.1016/0006-3223(92)90097-J 1511075

[B44] Mesquita A. R. Correia-Neves M. Roque S. Castro A. G. Vieira P. Pedrosa J. (2008). IL-10 modulates depressive-like behavior. J. Psychiatr. Res. 43, 89–97 10.1016/j.jpsychires.2008.02.004 18394646

[B45] Milad M. R. Pitman R. K. Ellis C. B. Gold A. L. Shin L. M. Lasko N. B. (2009). Neurobiological basis of failure to recall extinction memory in posttraumatic stress disorder. Biol. Psychiatry. 66, 1075–1082 10.1016/j.biopsych.2009.06.026 19748076 PMC2787650

[B46] Mouzon B. Chaytow H. Crynen G. Bachmeier C. Stewart J. Mullan M. (2012). Repetitive mild traumatic brain injury in a mouse model produces learning and memory deficits accompanied by histological changes. J. Neurotrauma 29, 2761–2773 10.1089/neu.2012.2498 22900595 PMC13588329

[B47] Mouzon B. C. Bachmeier C. Ferro A. Ojo J. O. Crynen G. Acker C. M. (2013). Chronic neuropathological and neurobehavioral changes in a repetitive mild traumatic brain injury model. Ann. Neurol. 75, 241–254 10.1002/ana.24064 24243523

[B48] Norrholm S. D. Jovanovic T. Olin I. W. Sands L. A. Karapanou I. Bradley B. (2011). Fear extinction in traumatized civilians with posttraumatic stress disorder: relation to symptom severity. Biol. Psychiatry 69, 553–563 10.1016/j.biopsych.2010.09.013 21035787 PMC3052965

[B49] Ohry A. Rattok J. Solomon Z. (1996). Post-traumatic stress disorder in brain injury patients. Brain Inj. 10, 687–695 10.1080/026990596124106 8853871

[B50] Ojo B. Rezaie P. Gabbott P. L. Cowely T. R. Medvedev N. I. Lynch M. A. (2011). A neural cell adhesion molecule-derived peptide, FGL, attenuates glial cell activation in the aged hippocampus. Exp. Neurol. 232, 318–328 10.1016/j.expneurol.2011.09.025 21978973

[B51] Ojo J. O. Mouzon B. Greenberg M. B. Bachmeier C. Mullan M. Crawford F. (2013). Repetitive mild traumatic brain injury augments tau pathology and glial activation in aged hTau mice. J. Neuropathol. Exp. Neurol. 72, 137–151 10.1097/NEN.0b013e3182814cdf 23334597

[B52] Omalu B. Hammers J. L. Bailes J. Hamilton R. L. Kamboh M. I. Webster G. (2011). Chronic traumatic encephalopathy in an Iraqi war veteran with posttraumatic stress disorder who committed suicide. Neurosurg. Focus 31, E3 10.3171/2011.9.FOCUS11178 22044102

[B53] Park C. R. Zoladz P. R. Conrad C. D. Fleshner M. Diamond D. M. (2008). Acute predator stress impairs the consolidation and retrieval of hippocampus-dependent memory in male and female rats. Learn. Mem. 15, 271–280 10.1101/lm.721108 18391188 PMC2327269

[B54] Pitman R. K. Orr S. P. (1990). Twenty-four hour urinary cortisol and catecholamine excretion in combat-related posttraumatic stress disorder. Biol. Psychiatry 27, 245–247 10.1016/0006-3223(90)90654-K 2294983

[B55] Povlishock J. T. (1993). Pathobiology of traumatically induced axonal injury in animals and man. Ann. Emerg. Med. 22, 980–986 10.1016/S0196-0644(05)82738-6 8503536

[B56] Rand C. W. Courville C. B. (1946). Histologic changes in the brain in cases of fatal injury to the head; alterations in nerve cells. Arch. Neurol. Psychiatry 55, 79–110 10.1001/archneurpsyc.1946.02300130003001 21016913

[B57] Rauch S. L. Shin L. M. Phelps E. A. (2006). Neurocircuitry models of posttraumatic stress disorder and extinction: human neuroimaging research–past, present, and future. Biol. Psychiatry 60, 376–382 10.1016/j.biopsych.2006.06.004 16919525

[B58] Rohleder N. Joksimovic L. Wolf J. M. Kirschbaum C. (2004). Hypocortisolism and increased glucocorticoid sensitivity of pro-inflammatory cytokine production in Bosnian war refugees with posttraumatic stress disorder. Biol. Psychiatry 55, 745–751 10.1016/j.biopsych.2003.11.018 15039004

[B59] Sandi C. Pinelo-Nava M. T. (2007). Stress and memory: behavioral effects and neurobiological mechanisms. Neural Plast. 2007:78970 10.1155/2007/78970 18060012 PMC1950232

[B60] Schneiderman A. I. Braver E. R. Kang H. K. (2008). Understanding sequelae of injury mechanisms and mild traumatic brain injury incurred during the conflicts in Iraq and Afghanistan: persistent postconcussive symptoms and posttraumatic stress disorder. Am. J. Epidemiol. 167, 1446–1452 10.1093/aje/kwn068 18424429

[B61] Seedat S. Stein M. B. Kennedy C. M. Hauger R. L. (2003). Plasma cortisol and neuropeptide Y in female victims of intimate partner violence. Psychoneuroendocrinology 28, 796–808 10.1016/S0306-4530(02)00086-0 12812865

[B61a] Shin L. M. Liberzon I. (2010). The neurocircuitry of fear, stress, and anxiety disorders. Neuropsychopharmacology 35, 169–191 10.1038/npp.2009.83 19625997 PMC3055419

[B62] Smith D. H. Lowenstein D. H. Gennarelli T. A. McIntosh T. K. (1994). Persistent memory dysfunction is associated with bilateral hippocampal damage following experimental brain injury. Neurosci. Lett. 168, 151–154 10.1016/0304-3940(94)90438-3 8028769

[B63] Spivak B. Shohat B. Mester R. Avraham S. Gil-Ad I. Bleich A. (1997). Elevated levels of serum interleukin-1 beta in combat-related posttraumatic stress disorder. Biol. Psychiatry 42, 345–348 10.1016/S0006-3223(96)00375-7 9276074

[B64] Stein M. B. Yehuda R. Koverola C. Hanna C. (1997). Enhanced dexamethasone suppression of plasma cortisol in adult women traumatized by childhood sexual abuse. Biol. Psychiatry 42, 680–686 10.1016/S0006-3223(96)00489-1 9325561

[B65] Tanriverdi F. Unluhizarci K. Kelestimur F. (2010). Pituitary function in subjects with mild traumatic brain injury: a review of literature and proposal of a screening strategy. Pituitary 13, 146–153 10.1007/s11102-009-0215-x 20037793

[B66] van der Kolk B. A. Fisler R. (1995). Dissociation and the fragmentary nature of traumatic memories: overview and exploratory study. J. Trauma. Stress 8, 505–525 10.1002/jts.2490080402 8564271

[B67] Vanderploeg R. D. Belanger H. G. Horner R. D. Spehar A. M. Powell-Cope G. Luther S. L. (2012). Health outcomes associated with military deployment: mild traumatic brain injury, blast, trauma, and combat associations in the Florida National Guard. Arch. Phys. Med. Rehabil. 93, 1887–1895 10.1016/j.apmr.2012.05.024 22705240

[B68] Vasterling J. J. Brailey K. Constans J. I. Sutker P. B. (1998). Attention and memory dysfunction in posttraumatic stress disorder. Neuropsychology 12, 125–133 10.1037/0894-4105.12.1.125 9460740

[B69] Vasterling J. J. Brailey K. Proctor S. P. Kane R. Heeren T. Franz M. (2012). Neuropsychological outcomes of mild traumatic brain injury, post-traumatic stress disorder and depression in Iraq-deployed US Army soldiers. Br. J. Psychiatry 201, 186–192 10.1192/bjp.bp.111.096461 22743844

[B70] Vasterling J. J. Verfaellie M. Sullivan K. D. (2009). Mild traumatic brain injury and posttraumatic stress disorder in returning veterans: perspectives from cognitive neuroscience. Clin. Psychol. Rev. 29, 674–684 10.1016/j.cpr.2009.08.004 19744760

[B71] Warden D. L. Labbate L. A. Salazar A. M. Nelson R. Sheley E. Staudenmeier J. (1997). Posttraumatic stress disorder in patients with traumatic brain injury and amnesia for the event? J. Neuropsychiatry Clin. Neurosci. 9, 18–22 9017524 10.1176/jnp.9.1.18

[B72] Witgen B. M. Lifshitz J. Smith M. L. Schwarzbach E. Liang S. L. Grady M. S. (2005). Regional hippocampal alteration associated with cognitive deficit following experimental brain injury: a systems, network and cellular evaluation. Neuroscience 133, 1–15 10.1016/j.neuroscience.2005.01.052 15893627

[B73] Woodcock T. Morganti-Kossmann M. C. (2013). The role of markers of inflammation in traumatic brain injury. Front. Neurol. 4:18 10.3389/fneur.2013.00018 23459929 PMC3586682

[B74] Yehuda R. Boisoneau D. Lowy M. T. Giller E. L. Jr. (1995a). Dose-response changes in plasma cortisol and lymphocyte glucocorticoid receptors following dexamethasone administration in combat veterans with and without posttraumatic stress disorder. Arch. Gen. Psychiatry 52, 583–593 10.1001/archpsyc.1995.03950190065010 7598635

[B75] Yehuda R. Kahana B. Binder-Brynes K. Southwick S. M. Mason J. W. Giller E. L. (1995b). Low urinary cortisol excretion in Holocaust survivors with posttraumatic stress disorder. Am. J. Psychiatry 152, 982–986 7793468 10.1176/ajp.152.7.982

[B76] Young E. A. Breslau N. (2004). Cortisol and catecholamines in posttraumatic stress disorder: an epidemiologic community study. Arch. Gen. Psychiatry 61, 394–401 10.1001/archpsyc.61.4.394 15066898

[B77] Yuan X. Q. Wade C. E. (1991). Neuroendocrine abnormalities in patients with traumatic brain injury. Front. Neuroendocrinol. 12, 209–230 11538874

[B78] Zetterberg H. Smith D. H. Blennow K. (2013). Biomarkers of mild traumatic brain injury in cerebrospinal fluid and blood. Nat. Rev. Neurol. 9, 201–210 10.1038/nrneurol.2013.9 23399646 PMC4513656

[B79] Zoladz P. R. Conrad C. D. Fleshner M. Diamond D. M. (2008). Acute episodes of predator exposure in conjunction with chronic social instability as an animal model of post-traumatic stress disorder. Stress 11, 259–281 10.1080/10253890701768613 18574787 PMC2535807

[B80] Zoladz P. R. Diamond D. M. (2013). Current status on behavioral and biological markers of PTSD: a search for clarity in a conflicting literature. Neurosci. Biobehav. Rev. 37, 860–895 10.1016/j.neubiorev.2013.03.024 23567521

[B81] Zoladz P. R. Fleshner M. Diamond D. M. (2012). Psychosocial animal model of PTSD produces a long-lasting traumatic memory, an increase in general anxiety and PTSD-like glucocorticoid abnormalities. Psychoneuroendocrinology 37, 1531–1545 10.1016/j.psyneuen.2012.02.007 22421563

